# The Cs_2_AgRhCl_6_ Halide Double Perovskite: A Dynamically Stable Lead-Free Transition-Metal Driven Semiconducting Material for Optoelectronics

**DOI:** 10.3389/fchem.2020.00796

**Published:** 2020-10-28

**Authors:** Pradeep R. Varadwaj, Helder M. Marques

**Affiliations:** ^1^Department of Chemical System Engineering, School of Engineering, The University of Tokyo, Tokyo, Japan; ^2^Molecular Sciences Institute, School of Chemistry, University of the Witwatersrand, Johannesburg, South Africa

**Keywords:** *A*_2_AgRhCl_6_ halide double perovskites, first-principles studies, optoelectronic properties, geometrical, dynamical and mechanical stabilities, DOS and band structures

## Abstract

*A*-Site doping with alkali ions, and/or metal substitution at the B and B′-sites, are among the key strategies in the innovative development of *A*_2_BB′X_6_ halide double perovskite semiconducting materials for application in energy and device technologies. To this end, we have investigated an intriguing series of five halide-based non-toxic systems, *A*_2_AgRhCl_6_ (*A* = Li, Na, K, Rb, and Cs), using density functional theory at the SCAN-*rVV*10 level. The lattice stability and bonding properties emanating from this study of *A*_2_AgRhCl_6_ matched well with those that have already been synthesized, characterized and discussed [viz. Cs_2_AgBiX_6_ (X = Cl, Br)]. Exploration of traditional and recently proposed tolerance factors has enabled us to identify *A*_2_AgRhCl_6_ (*A* = K, Rb and Cs) as stable double perovskites. The band structure and density of states calculations suggested that the electronic transition from the top of the valence band [Cl(3p)+Rh(4d)] to the bottom of the conduction band [(Cl(3p)+Rh(4d)] is inherently direct at the X-point of the first Brillouin zone. The (non-spin polarized) bandgap of these materials was found in the range 0.57–0.65 eV with SCAN-*rVV*10, which were substantially smaller than those computed with hybrid HSE06 and PBE0, and quasi-particle GW methods. This, together with the appreciable refractive index and high absorption coefficient in the region covering the range 1.0–4.5 eV, enabled us to demonstrate that *A*_2_AgRhCl_6_ (*A* = K, Rb, and Cs) are likely candidate materials for photoelectric applications. The results of our phonon calculations at the harmonic level suggested that the Cs_2_AgRhCl_6_ is the only system that is dynamically stable (no imaginary frequencies found around the high symmetry lines of the reciprocal lattice), although the elastic moduli properties suggested all five systems examined are mechanically stable.

**Graphical Abstract:**
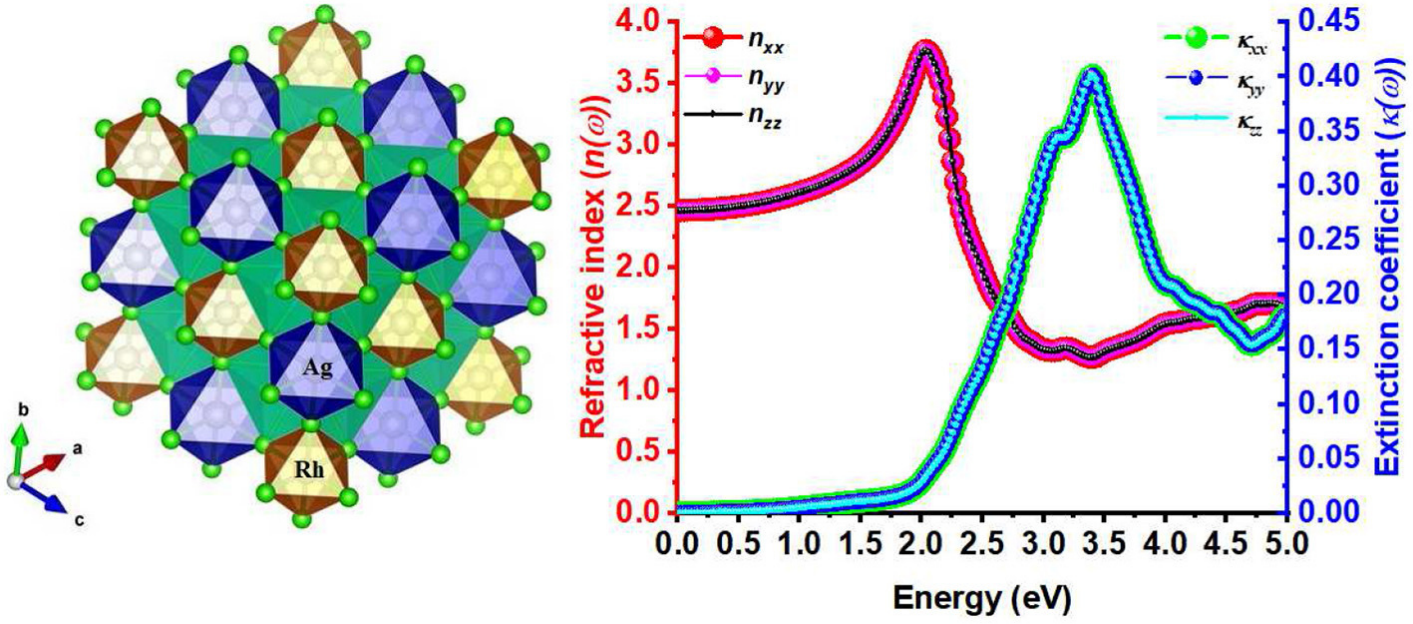
DFT modeling of the Cs_2_AgRhCl_6_ halide double perovskite (left) predicts a mechanically and dynamically stable material with reasonably high indices of refraction (right), suggesting possible application in optoelectronics.

## Introduction

Dynamically and Mechanically Stable Halide Double Perovskites are an important class of light harvesting materials for application in solar energy technology and optoelectronics (Greul et al., 2017; Matthews et al., 2017; Xiao et al., 2017; Zhao X.-G. et al., 2017; Chen et al., 2018; Lei et al., 2018; Li H. et al., 2018; Li T. et al., 2018; Luo et al., 2018; Tan et al., 2018; Xu et al., 2018; Chu et al., 2019; Zhao et al., 2019; Zhou Y. et al., 2019). They are characterized by the chemical formula *A*_2_BB′X_6_, where *A* is generally a monocationic organic or alkali metal species such as MA^+^ (methyl ammonium), Cs^+^, Rb^+^; B is an alkali metal ion or a transition metal atom in its +1 oxidation state (for example, Cu^+^, Ag^+^, Na^+^); B′ is a transition or main group metal ion in the +3 oxidation state (In^3+^, Bi^3+^, Sb^3+^, Cr^3+^); and the X sites are occupied by halide ions. Examples of widely examined halide double perovskites include Cs_2_AgSbBr_6_ (Wei et al., 2019), Cs_2_AgBiX_6_ (Greul et al., 2017; Chen et al., 2018; Lei et al., 2018), Cs_2_CuInX_6_ (Zhao X.-G. et al., 2017), and Cs_2_AgInX_6_ (Volonakis et al., 2017), where *A* = Cs^+^, B = Ag^+^/Cu^+^, B′ = In^3+^, Bi^3+^, and X^−^ = Cl^−^, Br^−^, I^−^.

Many *A*_2_BB′X_6_ perovskites such as Cs_2_AgSbBr_6_ (Wei et al., 2019) and Cs_2_AgBiX_6_ (X = Cl, Br) (Mcclure et al., 2016) have been synthesized and their optoelectronic properties delineated. The great majority of them exhibited indirect bandgap transitions so they are not ideal for thin film photovoltaic applications (Mcclure et al., 2016; Volonakis et al., 2017; Zhao X.-G. et al., 2017; Wei et al., 2019). Many with direct bandgaps were also synthesized, but the first onset of optical absorption for several of them was beyond what might be expected from the Shockley–Queisser (S-Q) limit (Shockley and Queisser, 1961). For instance, Cs_2_InAgCl_6_ has a direct bandgap of 3.3 eV, in which the first onset of optical absorption was observed at 380 nm, with a second absorption at 585 nm (Volonakis et al., 2017); this is therefore not suitable for application in a solar cell because the S-Q limit suggests that the maximum theoretical efficiency of a solar cell can be achieved with materials that, among other properties, exhibit direct bandgaps between 1.1 and 1.4 eV (Rühle, 2016).

The search for ideal candidate materials for photovoltaics can be achieved through *A*, B, and B′-site doping (Yang et al., 2018) and this has already led to the discovery of many direct bandgap 2D and 3D semiconducting materials that are environmentally friendly and stable (Slavney et al., 2018; Jana et al., 2019; Yao et al., 2020; Belding et al.,). Doping assists in changing the characteristic properties of the resulting materials by modifying, *inter alia*, the lattice parameters, cell volume, lattice density and bonding environments that manipulate the bandgap and the character of the valence band maximum (VBM) and the conduction band minimum (CBM) (Zhao et al., 2018; Wang Z. et al., 2019). Examples of such doped materials include the sodium-based perovskites Cs_2_NaB′X_6_ (B′ = Sb, Bi; X = Cl, Br, I) that produced the iodide perovskites Cs_2_NaSbI_6_ and Cs_2_NaBiI_6_, with optimal bandgaps of 2.03 and 2.43 eV, respectively. Since Cs_2_NaSbI_6_, Cs_2_NaBiI_6_, and Cs_2_NaSbBr_6_ exhibited appreciable absorption coefficients in the visible range, accompanied by p-p valence to conduction band transitions, these have been suggested as materials for solar cell applications (Zhao et al., 2018). Similarly, others (Yang et al., 2018) have observed that the bandgap of Cs_2_AgIn_*x*_Bi_1−x_Cl_6_ can be tuned from indirect (*x* = 0, 0.25, and 0.5) to direct (*x* = 0.75 and 0.9) by manipulating the percentage of doping, and that they exhibited 3 times greater absorption cross section, lower sub-bandgap trap states, and more than 5 times the photoluminescence quantum efficiency (PLQE) compared to those observed for indirect bandgap nanocrystals such as Cs_2_AgBiCl_6_. Bandgap tuning by alloying of Cs_2_AgBiCl_6_ nanocrystals resulted in a series of Cs_2_Na_*x*_Ag_1−x_BiCl_6_ (*x* = 0, 0.25, 0.5, 0.75, and 1) double perovskite nanocrystals that showed an increase in optical bandgap from 3.39 eV (*x* = 0) to 3.82 eV (*x* = 1) and a 30-fold increment in weak photoluminescence (Lamba et al., 2019). Other materials generated by replacing the B′-site species in *A*_2_BB′X_6_ with transition metals such as Mn^3+^ (Locardi et al., 2018; Nandha and Nag, 2018; Zhou J. et al., 2019), Cr^3+^ (Zhao et al., 2019), etc., via partial or heavy doping play a significant role in the discovery of innovative halide double perovskite materials for optoelectronics (Jain et al., 2017; Bartel et al., 2019; Cai et al., 2019; Li and Yang, 2019).

In this study, the electronic structures of a series of five double-halide perovskites *A*_2_AgRhCl_6_ (*A* = Li, Na, K, Rb, and Cs) are theoretically investigated using density functional theory at the SCAN-*rVV*10, PBE and PBEsol levels. We analyze their structural stability by means of the widely used octahedral and Goldsmith tolerance factors. An attempt is made to explore the same property using the Global Instability Index, as well as using a newly proposed tolerance factor, to demonstrate whether the traditionally-used octahedral and Goldsmith tolerance factors are adequate for identifying stable perovskites. The lattice constants, cell volume, cell density, density of states, and electronic structure properties are examined and discussed in light of the role that the *A*-site substitution (by the lighter alkali cations) plays in modifying the properties of Cs_2_AgRhCl_6_. The optical properties are investigated by calculating linear response characteristics such as the real and imaginary parts of the dielectric function, absorption coefficient, reflectivity and energy loss spectra. The reliability of electronic bandgaps of SCAN-*rVV*10 and those calculated using other GGA methods (GGA = Generalized Gradient Approximation) is assessed by comparing them with those calculated using HSE06, PBE0 and GW methods. The phonon modes, as well as the elastic properties, are calculated using density functional perturbation theory (DFPT) and finite difference method (FD) to probe the dynamical and mechanical stabilities, and the putative suitability of these materials for photovoltaic applications.

## Computational Details

The conventional unit-cell structures (lattice parameters, ionic positions and volumes, *etc*.) of *A*_2_AgRhCl_6_ (each comprises 40 atoms) were fully optimized using DFT. The same calculations were performed on their primitive unit cells (each comprises 10 atoms). The *k*-point mesh 8 × 8 × 8 centered at Γ was used for sampling the first Brillouin zone. The projector augmented wave (PAW) method (Blöchl, 1994), together with an energy cut-off of 520 eV for a plane wave basis set, was used. The equilibrium positions of the ions were calculated by structural optimization, where the internal degrees of freedom and lattice constants, along with the volume of the unit cell, were allowed to vary until the residual forces per atom were <0.006 eV/Å. The maximum and average forces acting on each ion were minimized to 0.006 and 0.004 eV/Å, respectively. Instead of a default value of 10^−4^, the allowed error in the total energy for relaxation of the electronic degrees of freedom was set to 10^−8^ eV. Calculations involving both spin and non-spin polarizations were performed.

The three different DFT functionals employed for the relaxation of the geometry of *A*_2_AgRhCl_6_ were SCAN-*rVV*10 (Sun et al., 2015; Sun J. et al., 2016; Buda et al., 2017), PBE (Perdew et al., 1996) and PBEsol (Perdew et al., 2008). The reason for choosing three functionals is that we were interested in determining the extent to which the latter two functionals underestimate the bandgaps of the systems under investigation compared to SCAN-*rVV*10, since they generally underestimate the bandgap of halide single and double perovskites compared to both experiment and the computationally expensive GW and HSE06 (Volonakis et al., 2017; Lamba et al., 2019; Umadevi and Watson, 2019; Wang H.-C. et al., 2019). We note that the newly-proposed SCAN-*rVV*10 functional is one of the strongly constrained and appropriately normed meta-generalized gradient approximation (meta-GGA) functionals that is considered to model well metallic, insulating and semiconducting materials (Sun et al., 2015; Sun J. et al., 2016; Buda et al., 2017). The *rVV*10 part of the functional accounts for the non-local correlation part required to appropriately describe van der Waals (vdW) interaction (Peng et al., 2016; Chakraborty et al., 2018; Zhang et al., 2018; Anh et al., 2019). Bokdam et al. have demonstrated that the SCAN functional accounts for short range dispersion effects—which conventional hybrid functionals do not account for—and is the most suitable functional to study the atomic structure of hybrid perovskite materials (Bokdam et al., 2017). The Vienna *Ab initio* Simulation Package (VASP) was used for all calculations (Kresse and Furthmüller, 1996a,b).

The tetrahedron method with Blöchl corrections was used for the calculation of the density of states (DOS) of *A*_2_AgRhCl_6_. Their electronic band structures were calculated using a standard Self-Consistent (SC) procedure, followed by a subsequent non-SC calculation (VASP, 2020e). The 15 × 15 × 15 *k*-point mesh was used for sampling the Brillouin zone and the primitive cells were used. The DOS and band structures of *A*_2_AgRhCl_6_ were plotted using Pyband (Qijingzheng) and Sumo (Ganose et al., 2018).

The optical properties, such as the real and imaginary parts of the frequency dependent dielectric function, were computed on the SCAN-*rVV*10 geometries using the PBEsol functional (Perdew et al., 2008), a functional that has been extensively used to calculate the linear response properties of halide perovskites (Brivio et al., 2013; Frost et al., 2014; Savory et al., 2016b; Jong et al., 2018). In these calculations, the number of empty conduction band states were doubled, together with the number of frequency grid points, which was set to 2000. The Density Functional Perturbation Theory (DFPT) method was adopted (Gonze, 1997; Gonze and Lee, 1997; Baroni et al., 2001; VASP, 2020b). The Γ-centered *k*-point meshes 8 × 8 × 8, 10 × 10 × 10, and 18 × 18 × 18, blocked Davidson iteration scheme, energy cut-off of 520 eV, and a tightly converged electronic wavefunction (within 10^−8^ eV) were used.

Although the SCAN-*rVV*10 functional was used for the calculation of lattice properties, density of states, and electronic band structures, it cannot be combined with DFPT for the evaluation of linear response (optical) properties due to its lack of implementation in VASP 5.4. For this reason, and for comparison purpose with the DFPT/PBEsol results, a separate set of calculations was performed using the meta-GGA functional using a Γ-centered *k*-point mesh 12 × 12 × 12 that invoked an electronic minimization algorithm for an exact diagonalization of the matrix, in which the derivative of the cell-periodic part of the orbitals w.r.t. **k**, |∇_**k**_u_n**k**_>, was calculated using finite differences given by Equation (1) (VASP, 2020c),

(1) $$|\nabla_{k}{\bar{u}}_{nk}\rangle =\sum_{n\neq n^{\prime }}\left|{\bar{u}}_{n^{\prime }k}\rangle \langle {\bar{u}}_{n^{\prime }k}\right|\frac{\partial [H(k)-\varepsilon_{nk}S(k)}{\varepsilon_{nk}-\varepsilon_{n^{\prime }k}}|{\bar{u}}_{nk}\rangle$$

where *H(****k****)* and *S(****k****)* are the Hamiltonian and overlap operator for the cell-periodic part of the orbitals, and the sum over *n*′ must include a sufficiently large number of unoccupied states.

The dynamical and mechanical stabilities of *A*_2_AgRhCl_6_ were examined using computed phonon band structures and elastic properties, respectively (Mouhat and Coudert, 2014; Togo and Tanaka, 2015; Kagdada et al., 2018). The former calculations were carried out using Phonopy (Togo and Tanaka, 2015). Both the DFPT/PBEsol and FD (Finite Difference) (Monserrat, 2018; VASP, 2020d) methods were used to calculate force constants in the reciprocal space. The 2 × 2 × 2 supercell structures (each 320 atoms) constructed using the conventional unit-cells of *A*_2_AgRhCl_6_ (*A* = Cs, Rb) (each 40 atoms) were supplied. The same practice was adopted to generate the supercell structures (each 80 atoms) using the primitive unit-cells of the system, and were used. Because the above calculations for systems with 320 atoms can be computationally very expensive, we used a 1 × 1 × 1 Γ-center scheme for *k*-point sampling integrations, together with an energy cut-off of 520 eV. For the latter supercells (80 atoms per supercell), a 4 × 4 × 4 *k*-mesh was used without changing other constraints.

The elastic coefficients (Mouhat and Coudert, 2014) of the stiffness matrix **C**_ij_ of *A*_2_AgRhCl_6_ (*A* = Cs, Rb, K, Li) were calculated within the harmonic approximation and finite differences to determine the second derivatives (Hessian matrix and phonon frequencies). A *k*-point mesh 6 × 6 × 6 was used. In all calculations referred to above, the SCAN-*rVV*10 optimized geometries of *A*_2_AgRhCl_6_ were used.

## Results and Discussion

### Geometrical Properties and Stability

The spin-polarized and spin non-polarized calculations gave very similar values for the total energies of each *A*_2_AgRhCl_6_. The calculated energy difference of the latter from the former (*per formula unit*) is −6.6, −7.3, −6.5, −5.2, and −3.6 meV for Cs_2_AgRhCl_6_, Rh_2_AgRhCl_6_, K_2_AgRhCl_6_, Na_2_AgRhCl_6_, and Li_2_AgRhCl_6_, respectively. Hence the spin-polarized systems were relatively more stable than the non-spin polarized systems. Unless otherwise stated, we report below the results of the most stable spin-polarized systems. We also confirm that the Rh^3+^ ions in *A*_2_AgRhCl_6_ had no local magnetic moments. Therefore, the chemical systems with perovskite stoichiometry examined in this work, *A*_2_AgRhCl_6_, contain low spin Rh^3+^ and are non-magnetic.

**Figure 1:**
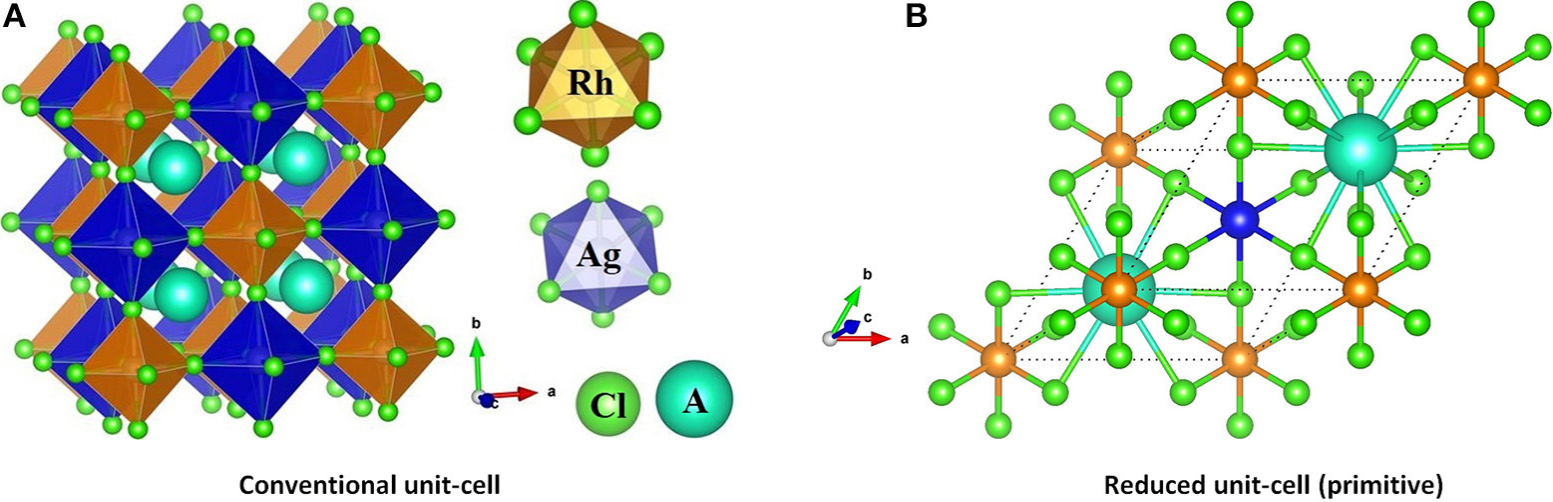
**(A)** SCAN-*rVV*10 relaxed polyhedral model of the conventional unit-cell of *A*_2_AgRhCl_6_, showing the coordination between the alkali metal anion and the halide anions. **(B)** The (reduced) primitive cell contains one unit of each of RhCl_6_ and AgCl_6_ octahedra in a face-centered cubic structure (space group *Fm*$\bar{3}$*m*).

The selected lattice properties of *A*_2_AgRhCl_6_ obtained with SCAN-*rVV*10 are given in Table 1; those calculated using PBE and PBEsol are given in Table S1. As expected, the lattice constants are equal, *a* = *b* = *c*, for each member of the series *A*_2_AgRhCl_6_. The largest value of the lattice constants found with SCAN-*rVV*10 was for Cs_2_AgRhCl_6_, 10.087 Å (Table 1); the decrease across the series studied correlates with the decrease in the ionic radius of the *A*-site cation. This is accompanied by a decrease in the metal–Cl and A–Cl bond distances and a contraction in cell volumes (Table 2). All the structures preserved a face-centered cubic symmetry (space group *Fm*$\bar{3}$*m*, Figure 1).

**Table 1:** Selected geometrical (lattice, volumetric, density, and stability) properties of *A*_2_AgRhCl_6_ (A = Cs, Rb, K, Na, Li) obtained with SCAN-*rVV*10.

**Compound**	***a* = *b* = *c* /Å**	**α = β = γ /deg**	**Volume/Å^**3**^**	**ρ /gcm^**−3**^**	***GII* /v.u**.
Cs_2_AgRhCl_6_	10.087	90	1026.2	4.46	0.121
Rb_2_AgRhCl_6_	9.960	90	988.0	4.00	0.148
K_2_AgRhCl_6_	9.898	90	968.6	3.44	0.170
Na_2_AgRhCl_6_	9.305	90	805.2	3.87	0.595
Li_2_AgRhCl_6_	9.803	90	941.9	3.08	0.423

*Conventional unit cells used*.

**Table 2:** Selected bond distances of *A*_2_AgRhCl_6_ (*A* = Li, Na, K, Rb, Cs).

**Compound**	***r*(Rh–Cl) /Å**	***r*(Ag–Cl) /Å**	***r*(*A*–Cl) /Å**
Cs_2_AgRhCl_6_	2.374	2.669	3.569
Rb_2_AgRhCl_6_	2.359	2.622	3.524
K_2_AgRhCl_6_	2.349	2.598	3.500
Na_2_AgRhCl_6_	2.252	2.400	3.290
Li_2_AgRhCl_6_	2.335	2.567	3.468

The PBE and PBEsol functionals predicted larger and smaller cell volumes and lattice constants compared to those of SCAN-*rVV*10, respectively (see Table S1). There is no experimental data available for these systems for comparison. To verify the reliability of SCAN-*rVV*10, we optimized the geometry of Cs_2_AgBiCl_6_ (and Cs_2_AgBiBr_6_) using the same theoretical method, in conjunction with the same *k*-point mesh and convergence criteria used for *A*_2_AgRhCl_6_ as detailed above. Our calculations gave a value of 10.667 Å (and 11.234 Å) for *a* (= *b* = *c*) for these two systems, which is in good agreement with the experimental value of 10.77687 Å (and 11.27123 Å) (Mcclure et al., 2016). The predicted (and experimental) cell volumes for Cs_2_AgBiCl_6_ and Cs_2_AgBiBr_6_ were 1210.33 Å^3^ (1251.6356 Å^3^) and 1417.87 Å^3^ (1431.904 Å^3^), respectively. We note that there is variability in the reported experimental cell volumes and lattice constants of these halide double perovskites, possibly due to variability in sample preparation and the experimental procedures adopted. For instance, Slavney et al. have reported experimental *a* and *V* values of 11.2499 and 1423.7901 Å^3^ for Cs_2_AgBiBr_6_ (Slavney et al., 2016), somewhat different to those reported by others (Mcclure et al., 2016; Zhou et al., 2017); the values we obtained were similar to those reported by Mcclure et al. (2016). This demonstrates that the predictability SCAN-*rVV*10 is better than that of PBE and PBEsol, and that this method is useful for the prediction of 3D crystal structures of related compounds.

Whether compounds of the type *A*BO_3_ (Li et al., 2004; Liu et al., 2008), *A*BX_3_ and *A*BB′X_6_ (Bartel et al., 2019) have a perovskite structure has often been judged based on the values of the octahedral factor μ and Goldschmidt tolerance factor, *t*, given by Equation (2):

(2) $$\begin{array}{r} \mu =\left(r_{\text{B}}/r_{X}\right);\;t{=}^{r_{A}+r_{\text{X}}}{/}_{\sqrt{2}\left(r_{B}+r_{\text{X}}\right)} \end{array}$$

For *A*BX_3_ and *A*BB′X_6_ perovskite crystals, 0.813 < *t* < 1.107 and 0.415 < μ < 0.895. Structures with μ and *t* values outside these ranges (as generally found for non-perovskites) cannot be regarded as stable perovskites, and those close to 1.0 are cubic. We used Shannon's radii (Shannon, 1976) for the ions in *A*_2_AgRhCl_6_ and for calculated μ and *t* (Table 3). The value of μ = 0.50 for *A*_2_AgRhCl_6_ is constant because *r*_X = Cl_ and *r*_B_ [B=(*r*_Ag_+*r*_Rh_)/2] are the same for all five members of the series. This, and the values calculated for *t*, suggest that the first four members of the series form stable perovskite structures. Cs_2_AgRhCl_6_ was recently recognized to form a double perovskite structure (μ = 0.50 and *t* = 0.96) (Bartel et al., 2019); we found that three other members of the series studied (Rb_2_AgRhCl_6_, K_2_AgRhCl_6_ and Na_2_AgRhCl_6_) may also form stable perovskite structures. However, with *t* = 0.71, Li_2_AgRhCl_6_ is unlikely to have a perovskite structure. Such a low *t* value is generally observed for significantly distored perovskites (viz. orthorhombic).

**Table 3:** Shannon's ionic radii (*r*) of ions, octahedral factor (μ), Goldschmidt tolerance factor (*t*), and new tolerance factor (τ) for *A*_2_AgRhCl_6_ (A = Cs, Rb, K, Na, Li).

**Compound**	***r_***A***_*/Å**	***r*[Ag^**+**^] /Å**	***r*[Rh^**3+**^] /Å**	***r_***B***_* = [[*r*(Ag^**+**^)+*r*(Rh^**3+**^)]/2] /Å**	**Cl^**−**^/Å**	***μ = r_***B***_/ r_***X***_***	***t***	**τ**
Cs_2_AgRhCl_6_	1.88	1.15	0.67	0.91	1.81	0.50	0.96	3.84
Rb_2_AgRhCl_6_	1.72	1.15	0.67	0.91	1.81	0.50	0.92	3.96
K_2_AgRhCl_6_	1.64	1.15	0.67	0.91	1.81	0.50	0.90	4.05
Na_2_AgRhCl_6_	1.39	1.15	0.67	0.91	1.81	0.50	0.83	4.59
Li_2_AgRhCl_6_	0.92	1.15	0.67	0.91	1.81	0.50	0.71	75.10

Other approaches, such as energy phase diagrams (Filip et al., 2018) and the global instability index (*GII*) (Salinas-Sanchez et al., 1992; Yamada et al., 2018) have been used for examining the feasibility of a compound adopting a perovskite structure. We have also used *GII* to shed more light on the probable stability of *A*_2_AgRhCl_6_ as perovskites, as *GII* is a measure of geometrical stability.

*GII* = 0.0 valence units (v.u.) for geometrically stable perovskite structures without steric distortions, and for empirically unstable structures, GII > 0.2 v.u. (Yamada et al., 2018). By definition, *GII* is the root mean square of the bond discrepancy index in the unit cell given by Equation (3),

(3) $$\begin{array}{r} GII=\sqrt{\sum_{i=1}^{N}d_{i}^{2}/N} \end{array}$$

where *N* is the number of ions, and *d* is the bond discrepancy factor. The latter is defined as the deviation of the bond valence sum (*BVS*) from the formal valence (Salinas-Sanchez et al., 1992; Yamada et al., 2018) which can be calculated using the sum of bond valences (*s*_*ij*_) around any specific ion. It is given by: $BVS=\sum_{i=1}^{n}s_{ij}$, where *s*_*ij*_ = exp((*l*_0_−*l*_*ij*_)/*b*), *l*_*ij*_ is a bond length, *l*_0_ is the bond valence parameter empirically determined using experimental room-temperature structural data, and *b* is the bond softness parameter. A detail of how this was done has been discussed in a number of previous studies (Brese and O'keeffe, 1991; Gagné and Hawthorne, 2015; Brown, 2017).

The *GII* values for *A*_2_AgRhCl_6_ (A = Cs, Rb, K, Na, Li) are given in Table 1. They are within the narrow range 0.12 v.u. < *GII* < 0.18 v.u. for *A* = Cs, Rb and K, but *GII* = 0.595 v.u. for *A* = Na and 0.423 v.u. for *A* = Li. This indicates that the first three are expected to form crystallographically stable perovskite structures, whereas the latter two are not. For comparison, SrTiO_3_, CaTiO_3_, NaTaO_3_, LaAlO_3_, and BaZrO_3_, were reported to have *GII* values of 0.006, 0.273, 0.102, 0.027, and 0.003 v.u., respectively, and crystallized into cubic perovskite structures under ambient conditions (Yamada et al., 2018).

There is no mutual agreement on whether *A*_2_AgRhCl_6_ will form a stable perovskite when using *GII* or the μ and *t* combination. This is unsurprising since *t* is not always a good predictor as it gives a high false-positive rate (51%) in the region of *t* where a perovskite is expected (0.825 < *t* < 1.059). Bartel et al. have tested a set of 576 ABX_3_ species and have found that *t* correctly predicted 94% of the known perovskites, but also 49% of the non-perovskites (Bartel et al., 2019). Because of this relatively poor predictability, these workers have proposed a new tolerance factor, τ, defined by Equation (4),

(4) $$\begin{array}{r} \tau =r_{X}/r_{\text{B}}-n_{A}\left(n_{A}-\left(r_{A}/r_{\text{B}}\right)/\ln\;\left(r_{A}/r_{\text{B}}\right)\right) \end{array}$$

where *n*_*A*_ is the oxidation state of *A*, *r*_i_ is the ionic radius of ion i, and *r*_*A*_ > *r*_*B*_ by definition. Although the second term of τ is different to *t*, the first term incorporates the octahedral term μ that manifests itself in the probability maps, particularly in the lower bound on *r*_*B*_ where perovskites are expected as *r*_*X*_ is varied. As *r*_*X*_ increases, *r*_*B*_ must similarly increase to enable the formation of stable BX_6_ octahedra. In particular, τ was shown to generalize outside the training set of 1034 experimentally observed single and double perovskites (91% accuracy) and was applied to identify 23,314 new double perovskites (A_2_BB′X_6_) ranked by their probability of being stable as perovskites based on the ranges of τ (τ < 4.18) that defines the decision boundary between a perovskite and a non-perovskite. Our results for τ are listed in Table 3; they indicate that *A*_2_AgRhCl_6_ (*A* = Cs, Rb, K) are stable perovskites (3.84 < τ < 4.05), but Na_2_AgRhCl_6_ (τ < 4.59) is only partially stable while Li_2_AgRhCl_6_ (τ < 75.10) is significantly unstable as a perovskite, and can be predicted to have a non-perovskite structure. These conclusions are in good agreement with the inferences drawn using *GII*.

It is worth mentioning that the coordination environment of Li^+^ in Li_2_AgRhCl_6_ is not remarkably different from that of the other *A*-site ions in the series *A*_2_AgRhCl_6_. The results of our calculations show that the alkali cations lie near the center of each cube in Figure 1 where they are involved in a dodecahedral arrangement with the coordinating Cl^−^ ions of *A*_2_AgRhCl_6_. This is evident in the data in Table 3; the Li–Cl bond distances are marginally smaller than those of the *A*–Cl (*A* = Cs, Rb, K) bond distances. The discrepancy in the trend in these distances between Li_2_AgRhCl_6_, Na_2_AgRhCl_6_ and K_2_AgRhCl_6_ is an artifact of the PAW potential in which the 3s and 2p semi-core states of Na were treated as valence states; consequently, the lattice constants and volume of Na_2_AgRhCl_6_ are predicted to be smaller than those of Li_2_AgRhCl_6_ (Table 1). It should also be noted that the indices μ, *t*, and τ were calculated using the ionic radii proposed by Shannon (1976), where the ionic radius of Li^+^ is for an 8-coordinate ion, whereas the ionic radii for the other ions are in a dodecahedral environment. This probably accounts for the very large τ value of 75.10 calculated for Li_2_AgRhCl_6_.

### Bandgap, Band Dispersion and Density of States Analyses

Cs_2_AgRhCl_6_ is a direct bandgap material, as are the other members of the series. The bandgap (*E*_g_) of Cs_2_AgRhCl_6_ with SCAN-*rVV*10 is 0.57 eV, indicating the possibility of electronic transition between the VBM and CBM. The *A*-site substitution in *A*_2_AgRhCl_6_ by lighter alkali atoms has a very small effect on the magnitude of *E*_g_, and were found to vary between 0.57 and 0.65 (Table 4 and Table S3). On the other hand, the PBEsol and PBE functionals gave *E*_g_ of 0.42 and 0.55 eV for Cs_2_AgRhCl_6_ (Table S1), respectively. This shows how these two functionals slightly underestimate *E*_g_ compared to the SCAN-*rVV*10 functional, while retaining the direct nature of the bandgap transition between the VBM and CBM.

**Table 4:** Non-spin-polarized effective masses of electrons and holes obtained using the parabolic fitting of the lower conduction band and upper valence band for *A*_2_AgRhCl_6_ (*A* = Cs, Rb, K, Na, Li)^a^. Primitive cells used.

**Compound**	**E_**g**_/eV**	**Nature of E_**g**_**	**Carrier type**	**Direction**			
				**X → Γ**	**X → W**	**Average**	ratio (|$m_{e}^{*}$/$m_{h}^{*}$|)
Cs_2_AgRhCl_6_	0.58	Direct at *X*	$m_{h}^{*}$/m_0_	−2.43	−1.44	−1.94	
			$m_{e}^{*}$/m_0_	0.56	0.35	0.45	0.23
Rb_2_AgRhCl_6_	0.62	Direct at *X*	$m_{h}^{*}$/m_0_	−1.73	−1.37	−1.55	
			$m_{e}^{*}$/m_0_	0.61	0.34	0.48	0.31
K_2_AgRhCl_6_	0.64	Direct at *X*	$m_{h}^{*}$/m_0_	−1.55	−1.35	−1.45	
			$m_{e}^{*}$/m_0_	0.64	0.34	0.49	0.34
Na_2_AgRhCl_6_	0.65	Direct at *X*	$m_{h}^{*}$/m_0_	−1.36	−1.28	−1.32	
			$m_{e}^{*}$/m_0_	0.66	0.34	0.50	0.38
Li_2_AgRhCl_6_	0.66	Direct at *X*	$m_{h}^{*}$/m_0_	−1.31	−1.25	−1.28	
			$m_{e}^{*}$/m_0_	0.67	0.34	0.50	0.39

a *m_0_ is the rest mass of the electron (9.11 × 10^−31^ kg)*.

**Figure 2:**
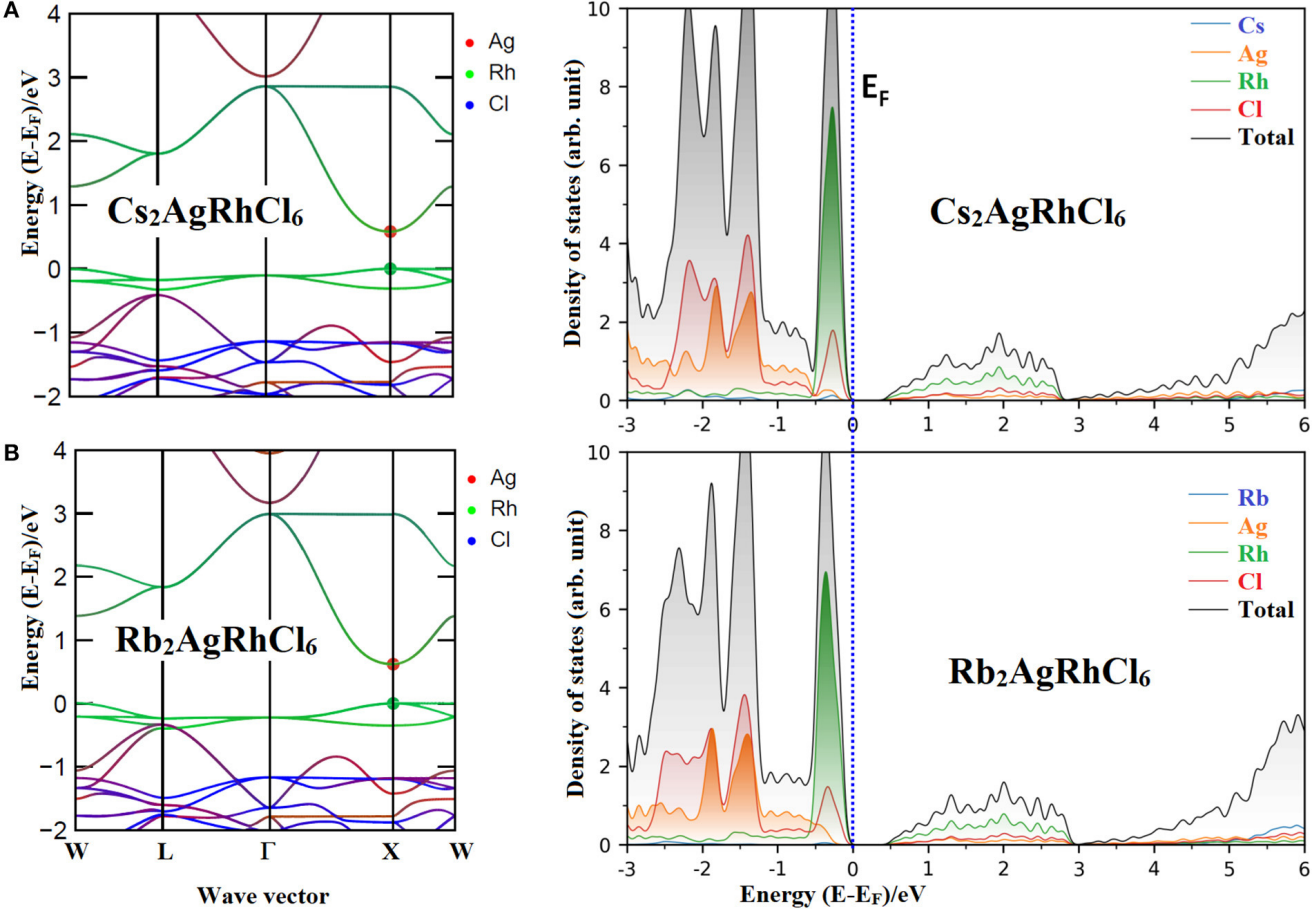
Comparison of the non-spin polarized atom-projected electronic band structure and partial (and total) density of states of **(A)** Cs_2_AgRhCl_6_ with **(B)** Rb_2_AgRhCl_6_. Different color codes were used for each plot. The *k*-vector types of space group *Fm*$\bar{3}$*m* were used for band structures.

From the plot of the density states (DOS) and electronic band structure of Cs_2_AgRhCl_6_ (Figures 2A,B, respectively), it was found that the VBM is of Cl(3p)–Rh (4d) character. In particular, the non-bonding orbital states t_2g_ (d_xy_, d_yz_, d_xz_) of Rh and the 3p orbital states of Cl cause the dispersion of the valence band just below the Fermi level. The calculated normalized contribution of Rh(4d) and Cl(3p) to the VBM are 68.4 and 28.5%, respectively. The contribution of the alkali and Ag atoms to the VBM of Cs_2_AgRhCl_6_ are negligibly small (1–2%).

By contrast, the edge associated with the CBM is largely derived from the Rh(4d) empty anti-bonding states e_g_ (d_z_2, d_x_2_−y_2), but the entire band is formed using contributions from Ag, Rh and Cl states leading to its dispersion far above the Fermi level. For example, the normalized contribution of Rh(4d), Cl(3p), and Ag(5s) to the CBM of Cs_2_AgRhCl_6_ were 61.6, 23.3, and 6.3%, respectively. These vary marginally upon the replacement of the *A*-site cation by lighter elements of the alkali group. For instance, the contribution of Rh(4d), Cl(3p), and Ag(5s) to the CBM of Rb_2_AgRhCl_6_ was 61.0, 23.5, and 6.0%, respectively, whereas those of Rb_2_AgRhCl_6_ were 60.8, 23.6, and 6.0%, respectively. Since the CBM is far away, and at the same time, the VBM is just below the fermi level, one might conclude that *A*_2_AgRhCl_6_ are p-type conducting materials (Wang H.-C. et al., 2019). The spin-polarized DOS and band structures of the first three members of the series are shown in Figures S1–S3, revealing that spin-polarization does not have any marked impact on the orbital character of CBM and VBM.

We note that the qualitative nature of orbital character responsible for the HOMO and LUMO bands do not change when the conventional cells of the corresponding systems were used for the same analysis. This suggests that the 4d orbitals of Rh^3+^ do indeed play a predominant role in driving the HOMO and LUMO bands of the studied systems (Figure S4A). However, the nature of the dispersion associated with the valence and conduction bands is significantly affected (Figure S4B). As can be seen, the band structure resembles the presence of parabolic double bands along the line *L* → Γ → *W* → *X* evaluated using the same band labels defined for the *Fm*$\bar{3}$*m* point group for a conventional cell (Bilbao Crystallographic Server). The bands are symmetric with respect to the mid-point of the Γ-*X* path, and are significantly flatter along Γ-*X-W* path that are associated with the valence band. These are clearly the effects of the double cell which can be thought as a supercell of the cubic halide perovskite. The flat bands indicate that there is no direct interaction between halide atoms along those directions, and the empty band dispersion shows that there is little interaction between Rh and the other atoms. It is therefore expected that the mobility of electrons associated with the conduction band edge should be faster than those of the holes at the valence band edge. Although this is reflected on the effective masses of the charge carriers discussed in the following section, it should be noted that the high-symmetry *k*-point paths are all defined for the primitive cell, but not for conventional cells. This explains why the origin of the bandgap transition is shifted from the *X*-point (Figure 2) to the center of the Brilliouin zone Γ-point (Figure S4B). Accordingly, the band structure shown in Figure S4A could be misleading; band unfolding is likely to recover the actual nature of the HOMO and LUMO bands that are apparent in Figure 2 and Figures S1–S3.

### Nature of Effective Masses and Their Mobility

We found that the top of the valence band is flatter than the bottom of the conduction band; the latter is appreciably parabolic. A similar flatter nature of the valence band was reported for bulk Cs_2_AgInCl_6_, which originated from Ag 4d and Cl 3p orbitals (Meng et al., 2017; Tran et al., 2017; Volonakis et al., 2017). In such a case, it is often observed that the hole effective mass (*m*_h_^*^) associated with the VBM is larger than the electron effective mass (*m*_e_^*^) of the CBM. Concomitantly, it is expected that the carrier mobility of the former is slower than that of the latter. The difference in the carrier masses arises from the nature of the curvature of the bottom of the conduction band (for electrons), or of the top of valence band (for holes); this is inversely proportional to the second derivative of the energy as a function of the wave vector **k**, and is described by the dispersion relationship shown in Equation (5),

(5) m*=±ħ2∂2E(k)∂k2

where the + and – signs refers electrons and holes, respectively (Hartmann et al., 1982; Opoku et al., 2017). Using parabolic fitting of the band edges, it was found that the spin-up holes and electrons are the dominant charge carriers. The VBM comprises two degenerate bands. One of them is heavier than the other. This is arguably due to the fact that one of these bands is very flat along *X* → *W* and other is parabolic (see Figures 2A,B). For the lighter HOMO band, the hole masses are heavier along the *X* → Γ direction compared to the *X* → *W* direction, a feature that is consistent in all five *A*_2_AgRhCl_6_ systems examined (Tables 4 and S3). The same behavior is associated with the effective masses of electrons that are virtually isotropic (values between 0.45 and 0.50 *m*_0_), suggesting that the charge transport would be predominant along the *X* → *W* direction.

**Figure 3:**
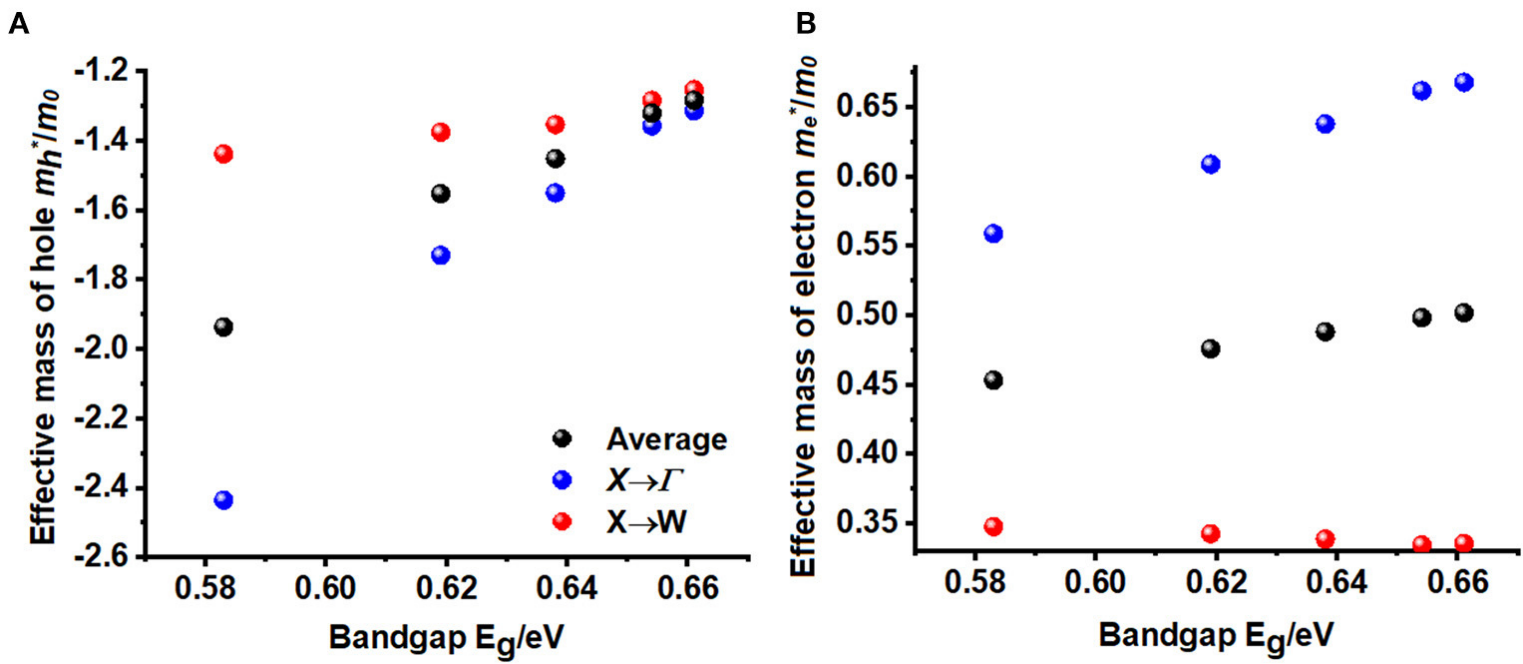
Dependence of non-spin polarized effective mass of **(A)** holes and **(B)** electrons holes on the bandgap E_g_ for *A*_2_AgRhCl_6_ (A = Cs, Rb, K, Na, Li).

The effective masses and bandgaps of *A*_2_AgRhCl_6_ obtained using non-spin polarized calculations were very similar to those calculated using the spin-polarized setting (see Table 4 and Table S3). Figure 3 illustrates the functional dependence of effective mass on the bandgap for the series *A*_2_AgRhCl_6_, which is independent of the nature of the spin- and non-polarized calculations performed. Nevertheless, the average effective mass of electrons is approximately 0.45 *m*_0_, whereas that of the hole is 1.94 *m*_0_ for Cs_2_AgRhCl_6_, where *m*_0_ is the free electron mass. These values are, respectively, 0.48 and 1.55 *m*_0_ for Rb_2_AgRhCl_6_; 0.49 and 1.45 *m*_0_ for K_2_AgRhCl_6_; 0.50 and 1.32 *m*_0_ for Na_2_AgRhCl_6_; and 0.50 and 1.28 *m*_0_ for Li_2_AgRhCl_6_. As mentioned above, the hole masses for one of the flatter valence bands should be heavier, reducing the average hole mobility. Indeed, this is what we have observed. It is smaller along the *X* → Γ direction than *X* → *W* direction (viz. 2.43 *m*_0_ vs. 27.0 *m*_0_); they are therefore are not incorporated in the average values shown in Table 4 and Table S3. A similar result was reported elsewhere for the Cs_2_InCuCl_6_ double perovskite (Pham et al., 2019).

These results not only suggest that the average spin-up holes are heavier than that of the spin-up electrons for *A*_2_AgRhCl_6_, but the relatively low electron effective mass also signifies the presence of n-type conductivity, as observed for the CdSe semiconductor, for example (Hartmann et al., 1982). Our result is consistent with Si, for which the electron mobilities are higher than hole mobilities. Since the hole mass for *A*_2_AgRhCl_6_ is comparable to that of other systems (Wang Z. et al., 2019), the contribution of the holes to charge transfer is unlikely to be very large (Park, 2019) and the lifetime of the charge carriers for these semiconductors is likely to be determined by electron-electron collisions (Kao, 2004; Morkoc, 2009; Fu and Zhao, 2018). The ratio $m_{e}^{*}$/*m*_h_^*^ provides the nature of electron-hole (e-h) pair stability in a recombination process (Zhang et al., 2012a,b; Dong et al., 2015; Faraji et al., 2015; De Lazaro et al., 2017; Opoku et al., 2017). In general, the larger the *m*${}_{e}^{*}$/m_h_^*^ ratio, the smaller the rate of recombination of the photoinduced charges. For instance, values of *m*${}_{e}^{*}$ and *m*_h_^*^ were found to be 0.24 *m*_0_ and 0.23 *m*_0_, respectively, for MAPbI_3_ (Filip et al., 2015). Similarly, they were 0.41 *m*_0_ (0.34 *m*_0_) and 0.35 *m*_0_ (0.37 *m*_0_) along the *R* to *X* direction for CsPbCl_3_ (CsPbBr_3_), respectively; 0.53 *m*_0_ (0.37 *m*_0_) along the *L* to *W* direction and 0.15 *m*_0_ (0.14 *m*_0_) along the *X* to Γ direction for Cs_2_AgBiCl_6_ (Cs_2_AgBiBr_6_), respectively (Mcclure et al., 2016). In all these cases, the ratio *m*${}_{e}^{*}$/m_h_^*^ is in the range 0.28 and 1.09, which is consistent with the majority of charge carriers in halide perovskites being large polarons; the slow recombination of these polarons underlies long carrier lifetime and diffusion length (Hoye et al., 2018; Zhang et al., 2019).

Our calculations gave $m_{h}^{*}$/m_e_^*^ values in the range between 2.6 (Li_2_AgRhCl_6_) and 4.4 (Cs_2_AgRhCl_6_), suggesting that these may be possible candidate materials for light-based device applications.

### Optical Properties

Insight into the optical properties of a solid state system can be obtained using the frequency dependent complex dielectric function ε(ω), a property that has been widely used to provide insight into, among other properties, the extent of charge screening, electron-hole coupling, and the electronic and ionic contributions to chemical bonding (Brivio et al., 2013; Walsh, 2015; Luo et al., 2017; Kirchartz et al., 2018; Wilson et al., 2019). The difference between the electronic and ionic dielectric constants (ε_ionic_ and ε_electronic_) assists to elucidate the polarity of the chemical bonds and the softness of the vibrations in a semiconducting material. The nature of the (picosecond) response of lattice vibrations (phonon modes) can be extracted, which can then be used to explain the extent of ionic and lattice polarizations required for a fundamental understanding of the photovoltaic performance of a material. If ε_1_ (ω) = $\varepsilon_{\alpha \beta }^{\left(1\right)}\left(\omega \right)$ is the real part, and ε_2_(ω) = $\varepsilon_{\alpha \beta }^{\left(2\right)}\left(\omega \right)$ is the imaginary part of the frequency dependent ε(ω), then ε(ω) can be written as ε(ω) = ε_1_(ω) + iε_2_(ω) = $\varepsilon_{\alpha \beta }^{\left(1\right)}\left(\omega \right)$+i$\varepsilon_{\alpha \beta }^{\left(2\right)}\left(\omega \right)$. We used the Kubo-Greenwood relationship (Equation 6) for the calculation of the frequency dependent dielectric matrix associated with $\varepsilon_{\alpha \beta }^{\left(2\right)}\left(\omega \right)$. This was determined upon summing over the empty states.

(6) $$\begin{array}{r} \varepsilon_{\alpha \beta }^{\left(2\right)}\left(\omega \right)=\frac{4\pi^{2}e^{2}}{\Omega }\lim_{q\to 0}\frac{1}{q^{2}}\underset{c,\upsilon ,k}{\sum }2w_{k}\delta \left(\varepsilon_{ck}-\varepsilon_{\upsilon k}-\omega \right)\\ \;\times \langle u_{ck+e\alpha q}|u_{\upsilon k}\rangle \langle u_{\upsilon k+e\beta q}|u_{\upsilon k}\rangle \end{array}$$

In Equation (6), the subscripts *c* and υ refer to conduction and valence band states, respectively, and *u*_*ck*_ is the cell periodic part of the orbitals at the k-point *k*. The real part $\varepsilon_{\alpha \beta }^{\left(1\right)}\left(\omega \right)$ of the dielectric function is related to $\varepsilon_{\alpha \beta }^{\left(2\right)}\left(\omega \right)$ via the Kramers-Kronig transformation given given by Equation (7), where *P* denotes the Cauchy principal value, and η is a small complex shift.

(7) $$\begin{array}{r} \varepsilon_{\alpha \beta }^{\left(1\right)}\left(\omega \right)=1+\frac{2}{\pi }P\overset{\propto }{\underset{0}{\int }}\frac{\varepsilon_{\alpha \beta }^{\left(2\right)}\left(\omega^{\prime }\right)\omega^{\prime }}{{\omega^{\prime }}^{2}-\omega^{2}+i\eta }d\omega^{\prime } \end{array}$$

**Figure 4:**
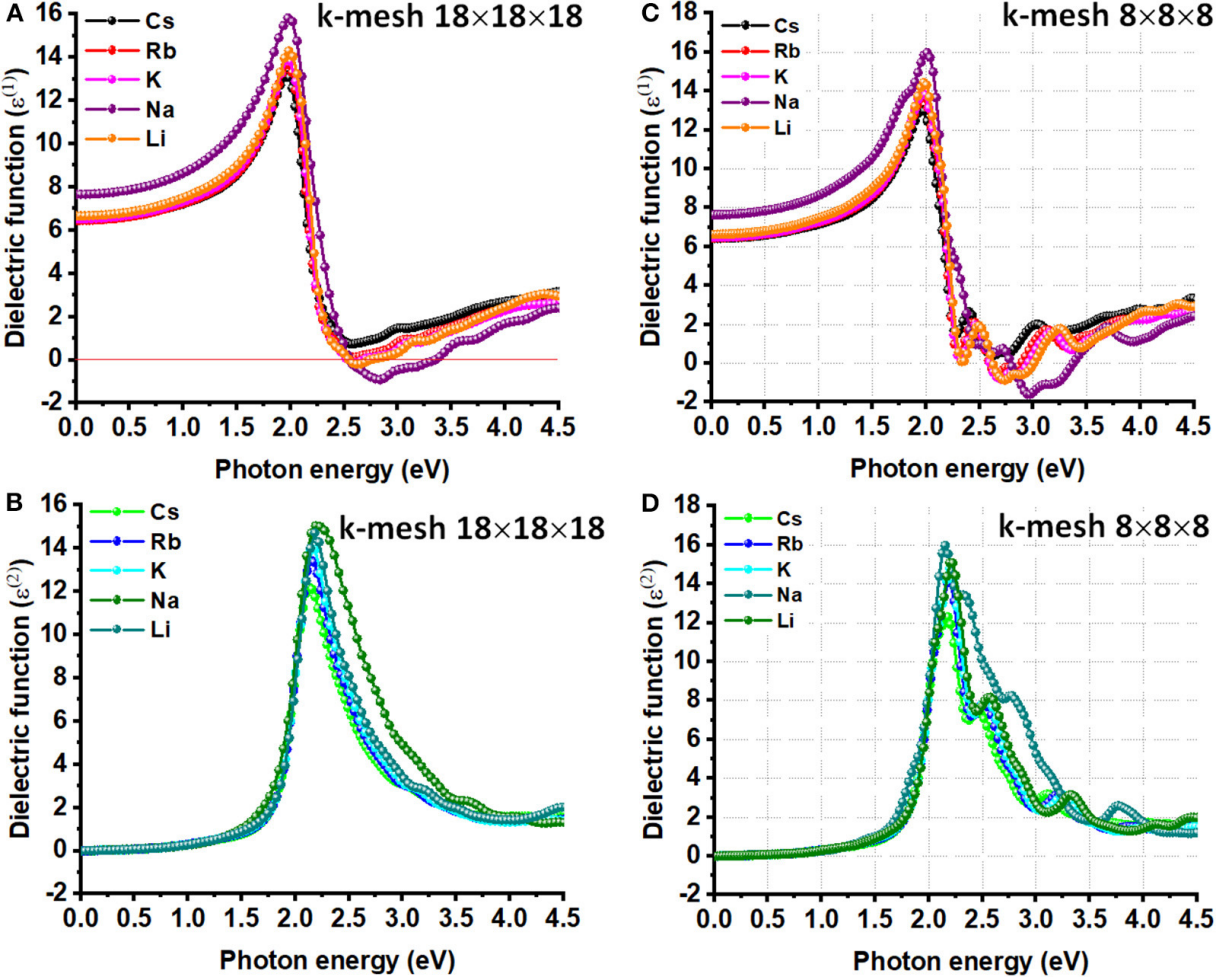
The **(A)** real and **(B)** imaginary parts of dielectric function ε(ω) plotted against the photon energy for *A*_2_AgRhCl_6_ (A = Li, K, Na, Rb, Cs), obtained using DFPT/PBEsol. Shown in **(C)** and **(D)** are the corresponding plots obtained using *k*-mesh 8x8x8. The primitive cells used.

The energy (or frequency) dependence of the dielectric function of *A*_2_AgRhCl_6_ in depicted in Figure 4. The curves of the real part of the dielectric function suggest that the electronic contribution to the static dielectric constant ε(0) is appreciably large, where ε(0) = ε_∞_ + ε_0_ (the first and last terms represent the electronic and ionic constributions, respectively). ε_∞_ is related to the vibrational polar phonons of the lattice (Yu, 2019), and is due to the (femtosecond) response of the electron density (Zangwill, 2019).

The ionic contribution, ε_0_, is due to the (picosecond) response of lattice vibrations (phonon modes); it is proportional to the polarity of the chemical bonds and the softness of the vibrations. The high frequency dielectric constant (ε_∞_), which is also called the optical dielectric constant, is found to be isotropic because of the cubic nature of each of the five systems examined. Its value is around 6.5 for Cs_2_AgRhCl_6_, which is also the zero-frequency limit of ε_1_(ω). This is the smallest across the series; of course, this is compared to the ε_∞_ values of 6.5, 6.5, 7.7, and 6.7 calculated for Rb_2_AgRhCl_6_, K_2_AgRhCl_6_, Na_2_AgRhCl_6_ and Li_2_AgRhCl_6_, respectively. The size of the *k*-mesh has a very marginal effect on the zero-frequency limit of of ε_1_(ω) (viz. Figure 4A vs. Figure 4C), regardless of the nature of the cell type used (Figure 4 vs. Figure S5). As noted above, the unusual trend in value of ε_∞_ for Na_2_AgRhCl_6_ may be a consequence of the plane wave basis set or the spin-polarized setting. The former could be corrected if the p-type semi-core states were to be treated as valence states, among other cut-off settings.

The ε_∞_ value of 6.5 found for Cs_2_AgRhCl_6_ is comparable to that reported for other absorbing materials (Wilson et al., 2019), e.g., CdTe (ε_∞_ = 7.1), MAPbI_3_ (ε_∞_ = 6.0), MAPbBr_3_ (ε_∞_ = 5.2), MAPbCl_3_ (ε_∞_ = 4.2) and CsPbI_3_ (ε_∞_ = 5.3). It is well-known that ε_∞_ is computationally sensitivity to the choice of electronic structure Hamiltonian and the level of geometry optimization; ε_∞_ values between 4.0 and 7.1 have been reported for the same material (MAPbI_3_), and experimentally determined to lie between 4.0 and 6.5 (Wilson et al., 2019). The experimental variability in ε_∞_ is either due to surface effects (Leguy et al., 2016a), or the assumptions taken in data processing (Hirasawa et al., 1994), or indeed other factors (Wilson et al., 2019). Using spectrospic ellipsometry methods, the optical dielectric constant was reported to be 4.8–5.1 near the absorption edge of CH_3_NH_3_PbBr_3_ organic-inorganic hybrid perovskite thin films, correspinding to a bandgap of 2.3 eV (Alias et al., 2016).

The maximum of ε_1_(ω) is approximately 13.0 for Cs_2_AgRhCl_6_. It shows up in the region between 0 and 4.5 eV. This becomes 13.7, 14.1, 15.5, and 14.3 for Rb_2_AgRhCl_6_, K_2_AgRhCl_6_, Na_2_AgRhCl_6_ and Li_2_AgRhCl_6_, respectively. The first corresponds to a photon energy of 2.0 eV, whereas those for others correspond to an energy around 2.0 eV. The use of smaller *k*-grid has some effect on the height of the peak, but not on the position of its occurrence (Figure 4A vs. Figure 4C). These results suggest that decreasing the size of the alkali metal ion in the lattice increases the high frequency response behavior, but the nature of the transitions involved between the VBM and CBM is of similar character.

On the other hand, the transition peaks in the curves of ε_2_(ω), which are directly related to the optical absorption process, occur at energies between 2.10 and 2.20 eV. These are located at higher energies than those in the ε_1_(ω) curves (see above). Whereas the ε_2_(ω) curves are quasi-symmetric and resemble a Lorentz-like resonant behavior that corresponds to bound electrons, the ε_1_(ω) curves are anti-symmetric and show dispersion-like behavior. The ε_1_(ω) spectra for K_2_AgRhCl_6_, Na_2_AgRhCl_6_, and Li_2_AgRhCl_6_ are strongly positive at low energies. It reaches a maximum value around 2.0 and then decreases. It becomes negative at the crossover energy around 2.5 eV and then becomes positive. The negative feature is likely to be a Drude-tail (Khatri et al., 2011; Eaton et al., 2018), thus indicating the quasi-metallic behavior of these materials in a specific region (Xu et al., 2008; Murtaza et al., 2011). This is not the case for Cs_2_AgRhCl_6_ and Rb_2_AgRhCl_6_. We note that the Drude feature appears in the ε_1_(ω) spectra using DFPT, which is persisent regardless of the size the *k*-mesh and cell-type used [Figure 4 and Figure S5 (Top)], is not evident in that calculated using SCAN-*rVV*10 (Figure S6), suggesting that it could be an artifact of DFPT.

The appreciable dielectric features delineated above are also evidence of the spectra of absorption coefficient α(ω), reflectivity *R*(ω), and energy loss function *L*(ω), calculated using Equations (8), (9), and (10), respectively (Dresselhaus, 2001; Ma et al., 2014), where c and ω are the speed of light in vacuum and frequency of light wave, respectively. The calculated real and imaginary parts of the dielectric function of each system were used.

(8) $$\begin{array}{r} \alpha \left(\omega \right)=\frac{\sqrt{2}\omega }{c}{\left\{{\left[\varepsilon_{1}^{2}\left(\omega \right)+\varepsilon_{2}^{2}\left(\omega \right)\right]}^{1/2}-\varepsilon_{1}\left(\omega \right)\right\}}^{1/2} \end{array}$$

(9) $$\begin{array}{r} R\left(\omega \right)={\left|\frac{\sqrt{\varepsilon_{1}\left(\omega \right)+i\varepsilon_{2}\left(\omega \right)}-1}{\sqrt{\varepsilon_{1}\left(\omega \right)+i\varepsilon_{2}\left(\omega \right)}+1}\right|}^{2}, \end{array}$$

(10) L(ω)=Im(-1ε(ω))=ε2(ω)ε12(ω)+iε22(ω)

**Figure 5:**
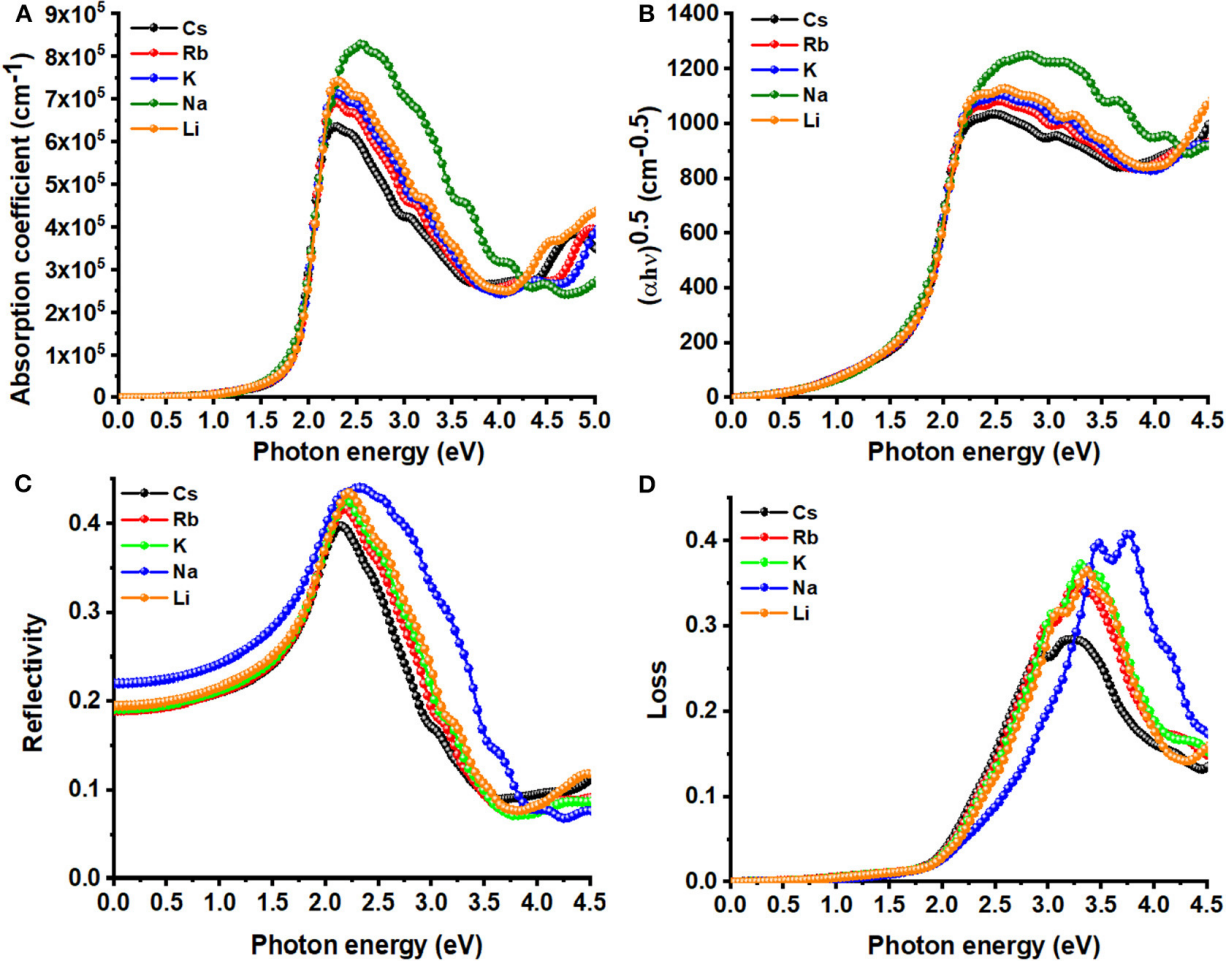
Dependence of the **(A)** absorption coefficient, **(C)** reflectivity, **(D)** energy-loss function on the photon energy of *A*_2_AgRhCl_6_ (*A* = Li, K, Na, Rb, Cs). Displayed in **(B)** is the Tauc plot for the corresponding systems. Primitive cells and *k*-mesh 18 × 18 × 18 used.

As noted elsewhere (Meng et al., 2017), absorption coefficients below 10^4^ cm^−1^ are regarded as a weak absorption; this may lead to a tail in the absorption coefficient curve of a UV-Vis spectrum. However, inspection of Figure 5A shows that the absorption starts around the VBM to the CBM transition region for all *A*_2_AgRhCl_6_. The absorption coefficient increases with an increase of photon energy, and reaches a maximum at 2.2 eV, which is in reasonable agreement with the maximum oscillator peak of ε_2_ as α is dependent on it (Equation 8). Since this occurs in the visible region, these systems could be useful in photovoltaic and photodetector applications (Wang et al., 2020). Specifically, the value of α at the highest peak varies between 6.4 × 10^5^ cm^−1^ (Cs_2_AgRhCl_6_) and 8.3 × 10^5^ cm^−1^ (Na_2_AgRhCl_6_); there should therefore be an appreciable absorption of light by these systems.

The computed absorption coefficient curves of *A*_2_AgRhCl_6_ show an onset around 1.1 and 1.3 eV; these are consistent with the SCF bandgaps predicted with SCAN-*rVV*10 (Table S2). The Tauc plot, Figure 5B, provides similar insight, since it is often used to extract the bandgaps from experimental absorption spectra (Eom et al., 2017; Tang et al., 2017; Habibi and Eslamian, 2018; Ji et al., 2018). The peak feature in the visible region with an appreciable absorption coefficient is a strong indication that *A*_2_AgRhCl_6_ are potential candidates for possible optoelectronic applications.

Since the VBM and CBM are substantially of Rh(4d) character, it is likely that the ligand field electronic transitions involved between them have d-d type metallic character (Ford, 2016). The Tanabe-Sugano diagram (Tanabe and Sugano, 1954) for a low spin d^6^ ion in an O_h_ environment indicates that the two lowest energy spin-allowed transitions from the ^1^A_1g_ state of the ion are, in order of increasing energy, ^1^A_1g_ → ^1^T_1g_ and ^1^A_1g_ → ^1^T_2g_. The spin-forbidden transitions ^1^A_1g_ → ^3^T_1g_ and ^1^A_1g_ → ^3^T_2g_ occur at longer wavelength. For instance, in some Rh^3+^-doped sodium borosilicate glasses thee spin-allowed transitions occur at 449 and 297 nm (2.76 and 4.17 eV, respectively) and the spin-forbidden transitions occur in the 600–2000 nm (2.0–0.62 eV) range (Wen et al., 2017). The emission onset and peak positions for Cs_2_NaMCl_6_:M' (M = Y, In, Sc; M′ = Rh^3+^) were reported between 1.3–1.4 and 1.04–1.07 eV, respectively, and ascribed to a ^1^A_1_ → ^3^T_1_ transition (Campochiaro et al., 1992). The observation of a peak emission at ~1,600 nm (0.77 eV) of the single luminescence band in the infrared spectral region in Rh^3+^-doped AgBr crystals was also ascribed to this spin-forbidden transition (Spoonhower et al., 1986).

We observe an absorption envelope beginning around the fundamental bandgap and expending into the visible and UV regions for *A*_2_AgRhCl_6_ (see Figures 5A). The three principal peaks were found around 2.2, 2.5, and 3.2 eV for Cs_2_AgRhCl_6_. The dominant absorption, around 2.2 eV, is attributed to the lower energy, spin-allowed transition (^1^A_1g_ → ^1^T_1g_); the higher energy spin-allowed transition (^1^A_1g_ → ^2^T_1g_) occurs around 3.2 eV. The lower energy shoulder on the main transition, around 2.5 eV, is ascribed to the spin-forbidden ^1^A_1g_ → ^3^T_1g_ transition. These transitions are prominent in the dielectric spectra shown in Figure 4D.

The complex refractive index (*n* +iκ) is a fundamental property of a solid material that describes the propagation velocity of light in the medium, thus allowing one to recognize whether such a material is potentially useful for optoelectronics (Schubert et al., 2007). The real and imaginary parts of the complex refractive index, called the static refractive index (*n(*ω*)*) and extinction coefficient (κ*(*ω*)*), were calculated using Equations (11) and (12), respectively (Dresselhaus, 2001; Li et al., 2009; Jong et al., 2016; Dong et al., 2017). Depending on the nature of the bulk material, the value of *n(*ω*)* varies, but κ*(*ω*)* is generally small for semiconductors (Baranoski and Krishnaswamy, 2010).

(11) $$\begin{array}{r} n\left(\omega \right)={\left[\frac{\sqrt{\varepsilon_{1}^{2}+\varepsilon_{2}^{2}}+\varepsilon_{1}}{2}\right]}^{1/2} \end{array}$$

(12) $$\begin{array}{r} \kappa \left(\omega \right)={\left[\frac{\sqrt{\varepsilon_{1}^{2}+\varepsilon_{2}^{2}}-\varepsilon_{1}}{2}\right]}^{1/2} \end{array}$$

**Figure 6:**
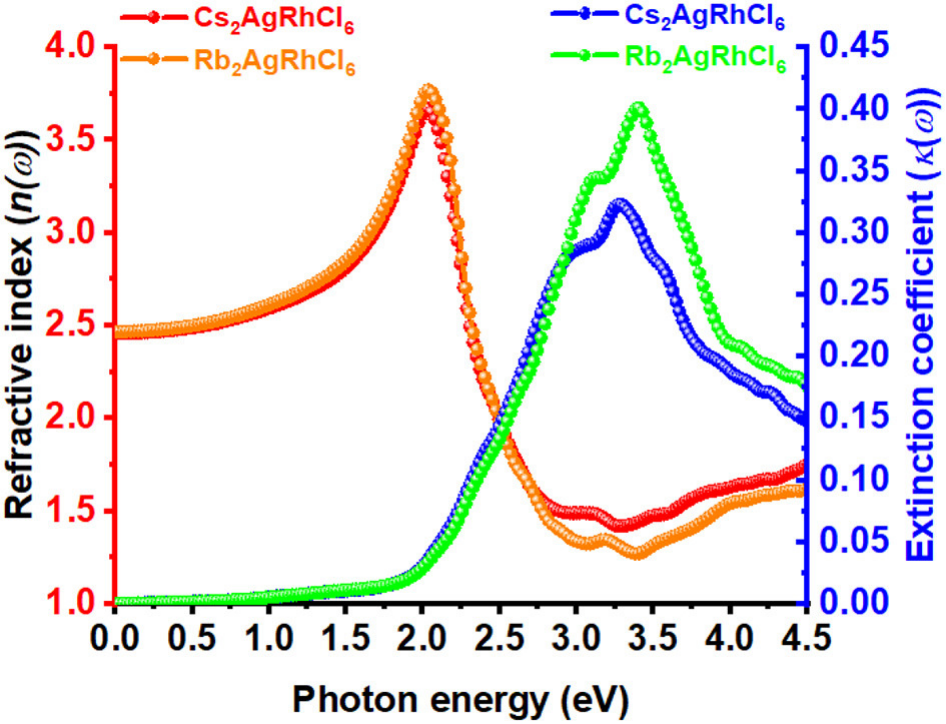
The dependence of the real and imaginary parts of the complex refractive index for Cs_2_AgRhCl_6_ and Rb_2_AgRhCl_6_. Although the corresponding spectra of *A*_2_AgRhCl_6_ (A = K, Na, Li) were of similar nature, they are not shown to avoid the complexity of the plot.

The static refractive index at the zero frequency limit of bulk Cs_2_AgRhCl_6_ is calculated to be 2.46, whereas that of Rb_2_AgRhCl_6_ is 2.47. Alkali introduction at the *A*-site caused a small increase in *n*, with values ranging between 2.46 and 2.50; this is caused by the contraction of the crystal lattice. As shown in Figure 6, *n(*ω*)* increases to a maximum value of 3.67 and 3.78 at the highest peaks, positioned at an energy of 2.03 eV for Cs_2_AgRhCl_6_ and Rb_2_AgRhCl_6_, respectively. Such large values of *n(*ω*)* are expected of optical ambient materials, with a typical refractive index of 2.5–3.5 (Schubert et al., 2007). For instance, the refractive index at the absorption edge was 2.29 and 2.61 for CH_3_NH_3_PbBr_3_ (Alias et al., 2016) and CH_3_NH_3_PbI_3_ (Löper et al., 2015) perovskites, respectively. CH_3_NH_3_PbI_3_ (Löper et al., 2015) as a single crystal and thin film, has a refractive index of 2.45 [at 800 nm (1.55 eV)] and 1.95, respectively (Löper et al., 2015).

The value of *n* for halide perovskites is significantly larger than that of SiO_2_ (1.09; Popova et al., 1972; Kitamura et al., 2007-−1.45; Malitson, 1965; Tan, 1998) or most polymers, making them a good material for resonant nanostructures due to their high optical contrast. Because *n* for Cs_2_AgRhCl_6_ is much lower than that of Si (*n* = 3.673; Refractiveindex.Info; Aspnes and Studna, 1983) or GaAs (3.4–3.7; Kachare et al., 1976; Aspnes et al., 1986; Jellison, 1992; Skauli et al., 2003), it provides high optical contrast with these materials in advanced hybrid structures (Makarov et al., 2019). By contrast, the extinction coefficient for Cs_2_AgRhCl_6_ is calculated to be very small and is close to 0.006 near the fundamental absorption edge of dispersion (Figure 6). This is comparable to the experimental κ values of 0.00014657, 0.08, and 0.005 reported for SiO_2_ (Popova et al., 1972; Kitamura et al., 2007), GaAs (Aspnes et al., 1986), and Si (Aspnes and Studna, 1983), respectively.

As can be seen from Figure 6, the values of the real and imaginary parts of the refractive index for each *A*_2_AgRhCl_6_ are substantially different to each other. Since reflectivity *R* is related to the refractive indices via *R* = ((*n*^2^ − κ^2^)/(*n*^2^ + κ^2^))^2^ and that *n* is several 100 orders of magnitude larger than κ at any specific critical point of the refractive index spectrum, it is clear that since *n* ≠ *k* the optical reflectivity will have a minimum value across the entire energy range investigated. This is seen in the reflectivity spectra calculated using the dielectric function (Equation 9), Figure 5C, in which a minimum reflection of <10% occurs in the region above 3.5 eV. The maximum of 38–45% light is likely to be reflected at the peak positions around 2.1 eV, and it is 15–25% around the onset of absorption. The reflectivity spectra of other systems reported elsewhere have similar characteristics (Peng et al., 2013; Ma et al., 2014; Saini et al., 2017; Mohamed et al., 2018). The energy loss function is plotted in Figure 5D. It is a measure of the loss of the energy of the electrons passing between energy bands. The maximum energy loss is calculated to lie between 32% (Cs_2_AgRhCl_6_) and 45% (Na_2_AgRhCl_6_) at energies of 3.2 and 3.6 eV, respectively.

Although the DFPT/PBEsol based optical properties described above were obtained using the primitive cells of *A*_2_AgRhCl_6_, the conventional cell geometries of these systems utilized for the same purpose using the same method did not significantly affect the location and height of peak features in the dielectric spectrum (Figure S5). However, when the same characteristics were investigated using SCAN-*rVV*10, it was found that although the nature of the transition features associated with the optical absorptions did not change markedly, the peak positions were blue-shifted. These were as large as 0.7–0.9 eV and the peak heights were reduced appreciably (Figure S6). In addition, the zero-frequency limit of ε^(1)^ of *A*_2_AgRhCl_6_, which were found between 6.5 and 8.0 (Figure 4A), is reduced, so they are predicted around 5.0 (Figure S6), yet confirming that the electronic contribution to the static dielectric constant is reasonably high.

Because of the appreciable inconsistency between the onsets of optical absorption in the dielectric spectra calculated using SCAN-*rVV*10 and DFPT/PBEsol, we extended our calculations to compute the bandgap of the studied systems using quasiparticle G_0_W_0_ and GW_0_ methods (Hedin, 1965; Van schilfgaarde et al., 2006; VASP, 2020a), based on Many-Body Perturbation Theory (MBPT), where G_0_ is one-particle Green's function, W_0_ is the screened Coulomb interaction, and GW_0_ is the most usual step beyond single-shot GW (G_0_W_0_) to iterate the quasi-particle energies in the Greens functions. The G_0_W_0_ approach calculated the Green's function G_0_ from the SCAN-*rVV*10 wavefunction in a non-self-consistent manner, and a perturbative scheme was utilized for the calculation of screened exchange in W_0_. In general, G_0_W_0_ calculates the quasi-particle energies from a single *GW* iteration by neglecting all off-diagonal matrix elements of the self-energy and employing a Taylor expansion of the self-energy around the DFT energies. The self-energy and one-shot calculations were performed using a 6 × 6 × 6 *k*-mesh. Interestingly, the SCAN-*rVV*10 predicted optical absorption edges associated with the imaginary part of the dielectric spectra (Figure S6) are in good agreement with the nature of the G_0_W_0_ and GW_0_ bandgaps of the corresponding systems. For instance, the G_0_W_0_ bandgaps were 2.43, 2.46 and 2.49 eV for Cs_2_AgRhCl_6_, Rb_2_AgRhCl_6_, and K_2_AgRhCl_6_ with the SCAN-*rVV*10 wavefunctions, respectively (Table S2). Similar calculations with the popular Heyd–Scuseria–Ernzerhof functional (HSE06) (Krukau et al., 2006; Savory et al., 2016a) and a 4 × 4 × 4 *k*-mesh gave bandgaps between 2.3 and 2.0 eV, showing an underestimation compared to G_0_W_0_ and GW_0_ (Table S2). Table S7 compares the lattice and bandgap properties of Cs_2_AgRhCl_6_ obtained from various computational approaches with those experimentally reported and calculated in this work for Cs_2_AgB′Cl_6_ (B' = In, Bi, Sb, Tl) (Zhou et al., 2018; Zhou J. et al., 2019).

The large difference between the GGA/meta-GGA and GW/HSE06 bandgaps is not very surprising given that the former ones generally underestimate bandgaps for single and double perovskite semiconductors (Ganose et al., 2017; Umadevi and Watson, 2019). For example, the reported PBE bandgap for Cs_2_AgInCl_6_ is 0.95 eV (Kumar et al., 2020), compared to the experimental value of 3.3 eV (Volonakis et al., 2017). Similarly, the bandgaps of 0.89 eV (PBEsol) and 1.1.17–1.61 eV (PBE) (Lu et al., 2016; Sun P.-P. et al., 2016; Zhao Y.-Q. et al., 2017) were reported for CH_3_NH_3_GeI_3_, and those of the hybrid functional range from 1.70 to 2.04 eV (Sun P.-P. et al., 2016; Zhao Y.-Q. et al., 2017), whereas the experimental value for which is 1.90 eV (Stoumpos et al., 2015). There is also precedence that the inclusion of dispersion correction further reduces the bandgaps, and this is likely the case with the GGA and HSE06 methods (Umadevi and Watson, 2019). The bandgaps found in the current study for *A*_2_AgRhCl_6_ are smaller/larger than, or comparable to, those of 2.19, 2.77, 2.33, and 3.00 eV reported for Cs_2_AgBiCl_6_, Cs_2_AgBiBr_6_, MAPbBr_3_ and MAPbCl_3_ (MA = methyl ammonium), respectively (Mcclure et al., 2016). There are other such double perovskite systems reported elsewhere (Jain et al., 2017; Zhao X.-G. et al., 2017; Locardi et al., 2018), which were stable and exhibited a direct bandgap in the spectral range relevant for solar energy conversion (1.5–2.5 eV), including Cs_2_AgInX_6_, Rb_2_AgInX_6_, and Rb_2_CuInX_6_ (X = Cl, Br). Clearly, the rhodium-based double perovskites examined in this study, which display excitonic features in the visible and UV regions, may be useful for applications in photoelectric detectors. MAPbBr_3_ is an exemplar photovoltaic semiconductor, with an experimental bandgap of 2.33 eV (Niemann et al., 2016; Varadwaj et al., 2018), which has been recognized as a material for photovoltaics and photodetectors (Saraf and Maheshwari, 2018).

### Phonon Features and Lattice Stability

**Figure 7:**
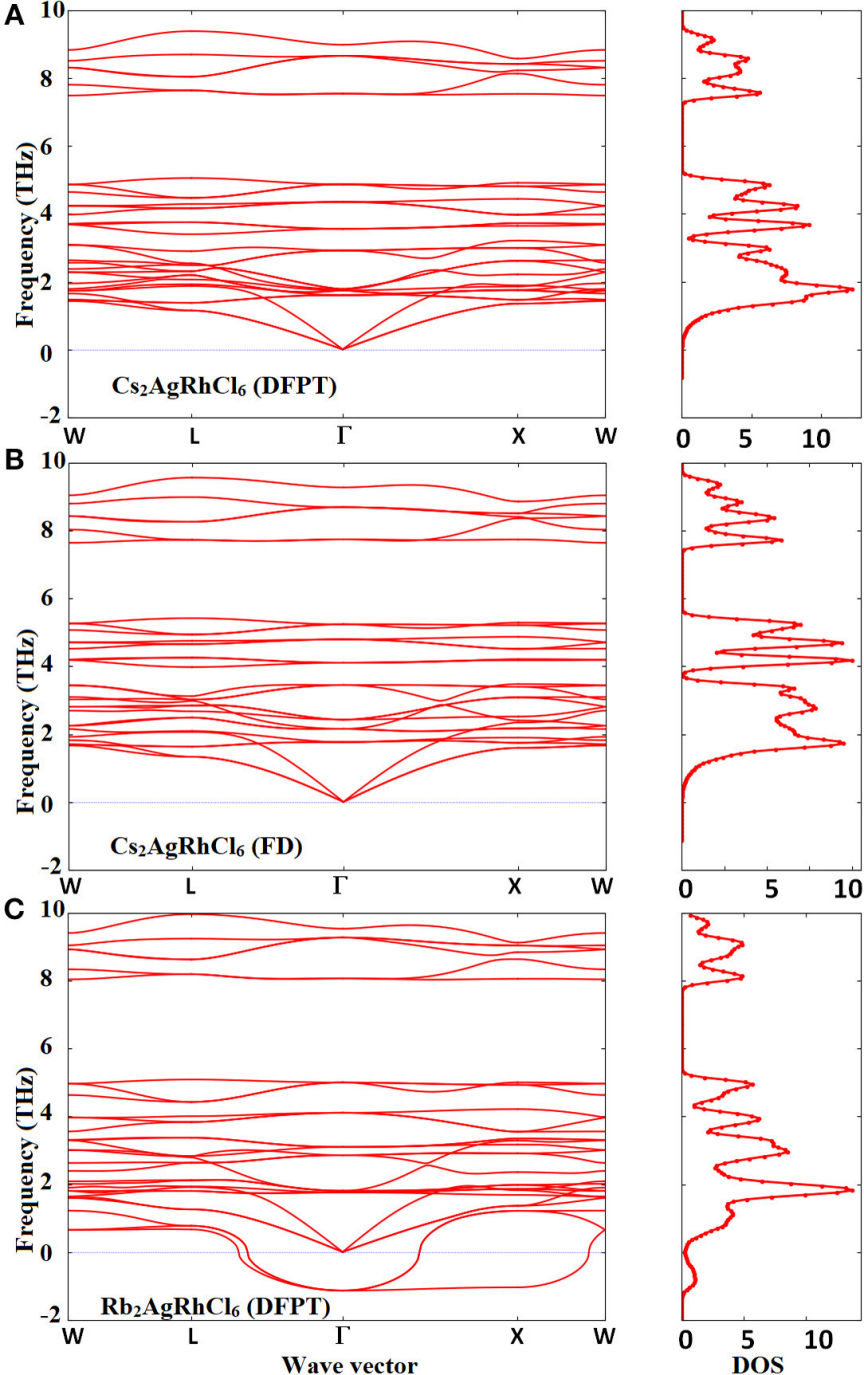
Comparison of the phonon dispersion and total phonon density of states (a.u.) of Cs_2_AgRhCl_6_ calculated using the **(A)** DFPT/PBEsol and **(B)** FD (finite difference) methods. Shown in **(C)** are the corresponding features for Rb_2_AgRhCl_6_, calculated using DFPT/PBEsol method with the supercell approach. Phonon “soft” modes are present around the Brillouin-zone boundary points and Γ → X → W for Rb_2_AgRhCl_6_.

The phonon band structures of Cs_2_AgRhCl_6_ and Rb_2_AgRhCl_6_ are illustrated in Figure 7 and Figure S7. The force constants defining the change in force on a reference atom in response to the displacement of another were used to construct a dynamics matrix. The matrix was then diagonalized. This gave the eigenvalues (normal mode phonon frequencies) and associated eigenvectors (phonon motion). As expected, there are three acoustic and several optical phonon modes. Although this is evidence of the dispersion curves shown in Figure S8, in which case, the conventional cells were used, two of these modes are found to be degenerate when the primitive cells used, showing the cell geometry plays an important role in unraveling the degeneracy involved. The acoustic modes are present below 1.5 THz and the optic modes are limited to the frequency interval 1.0–10.0 THz for Cs_2_AgRhCl_6_. These phonons are all stable across the Brillouin-zone boundary points, including the *W, L*, Γ, and *X*-points. This is true regardless of the nature of computational method employed (DFPT/PBEsol and FD, Figures 7A,B)—spin-polarized or non-spin polarized (see Figure S7). The only difference between the two theoretical approaches is that the phonon frequencies are somewhat different, with the FD method overestimating them compared to the DFPT/PBEsol method. However, all of them recognize the Cs_2_AgRhCl_6_ crystal lattice to be dynamically stable (Mouhat and Coudert, 2014).

For Rb_2_AgRhCl_6_ (Figure 7C), as well as the other three members of the *A*_2_AgRhCl_6_ series (not shown), the phonons are unstable along the entire path, especially along the paths *L* → Γ, Γ → *X* and *X* → *W*. They are associated with negative vibrational frequencies, which are due to the metastable lattice of the double perovskite induced by the alkali substitution at the *A*-site. The negative phonon modes are the so-called soft modes and are prominent, especially at the center of the Brillouin zone (Γ–point). This is evidence of the phonon density of states shown in Figure S9B (bottom), when compared against that shown in Figure S9A for Cs_2_AgRhCl_6_. The origin of such an instability for Rb_2_AgRhCl_6_ is probably a consequence of lattice softening induced by the softening of the shear constants for long wave phonons (Böni et al., 1988), and may not be ascribed to phonon instability that is generally caused by the softening of the transverse acoustic phonons near zone boundaries (Nakanishi et al., 1982; Liu et al., 2016). This is because the instability is primary associated with optical phonons, as the acoustic modes are unaffected. A somehow similar result was obtained for the other two members of the series, *A*_2_AgRhCl_6_ (*A* = Na, Li), but this is due to significant phonon and lattice instabilities tailored both by the acoustic phonons of low frequency and the optical modes of high frequency (not shown). Halide perovskites such as FAPbI_3_, MAPbI_3_, MAPbBr_3_, and CsPbBr_3_ were shown to exhibit phonon instabilities around the Brillouin-zone boundary points, which was significant at the *R*- and *M*-points; the imaginary frequency associated with the R-point was as large as 1.5 THz (for CsPbBr_3;_ Yang et al., 2017). This was attributed to the disorder introduced in the material by the anisotropic nature of the organic ion and octahedral tilting (Leguy et al., 2016b).

### Mechanical (Elastic) Properties

There are only three independent elastic constants (C_11_, C_12_, and C_44_) associated with the stiffness matrix C_*ij*_ of cubic crystals that represent the directional mechanical responses of the crystals for different directions of applied forces (Mouhat and Coudert, 2014). The longitudinal distortion, C_11_, is based on the longitudinal compression, and describes the hardness. The transverse distortion, C_12_, is based on the transverse expansion, which is related to Poisson's ratio. The shear elastic parameter, C_44_, is based on the shear modulus that represent the stiffness of the crystal. Our caclualtions gave all eigenvalues λ_i_ of the C_*ij*_ = C_*ji*_ matrix to be real and positive for all *A*_2_AgRhCl_6_ systems (Table S4). The Born criteria (Mouhat and Coudert, 2014) of the cubic system (i) C_11_ – C_12_ > 0; (ii) C_11_ + 2C_12_ > 0; (iii) C_44_ > 0 are also satisfied (see Table S5 for values of the elastic constants). This, along with the real and positive character of all the six eigenvalues of the stiffness matrix, points to the mechanical stability of all the five *A*_2_AgRhCl_6_ systems (Mouhat and Coudert, 2014).

The three stiffness constants are found in the order C_11_ > C_12_ > C_44_ (Table S5). The effect of the *A*-site cation on these constants is to increase C_11_ along the series (Cs_2_AgRhCl_6_ > Rb_2_AgRhCl_6_ > K_2_AgRhCl_6_ > Li_2_AgRhCl_6_), consistent with the decreasing ionic radii of the alkali ions. There was a concomitant decrease of C_12_ and C_44_ along the same line. This shows that alkali substitution at the *A*-site has a marked effect on C_11_ compared to C_12_ and C_44_; hence the longitudinal compression is increasingly larger compared to the transverse and shear distortions when passing from Cs^+^ through Rb^+^ to K^+^ to Li^+^. Because C_11_ ≠ C_12_ + 2C_44_, which is undoubtedly the result of significant longitudinal distortion, especially for systems containing Rb^+^, K^+^ and Li^+^, we conclude that *A*_2_AgRhCl_6_ (*A* = Rb, K, Li) are not strictly isotropic.

The shear constant C_44_, which measures plastic deformation, is less than (C_11_ – C_12_)/2 for each *A*_2_AgRhCl_6_. That is, the difference between them ([C_44_ – ((C_11_ – C_12_)/2)]) is negative (see Table S5). It increases from Cs_2_AgRhCl_6_ to Rb_2_AgRhCl_6_ to K_2_AgRhCl_6_ to Li_2_AgRhCl_6_, and hence the shear softening increases in the order Cs_2_AgRhCl_6_ < Rb_2_AgRhCl_6_ < K_2_AgRhCl_6_ < Li_2_AgRhCl_6_. This trend is roughly preserved for the shear modulus (see K values in Table S6), although the value of K for Cs_2_AgRhCl_6_ is slightly smaller than that for Rb_2_AgRhCl_6_ (15.47 GPa vs. 15.50 GPa). Clearly, the softening in the shear constant that results in very large negative values of [C_44_ – ((C_11_ – C_12_)/2)] (Table S4), except for Cs_2_AgRhCl_6_, probably explains the presence of the observed degenerate transverse branches shown in Figures 7A,B, and the lattice instability observed for Rb_2_AgRhCl_6_ and other members of the series (see Figure 7C and Figure S8).

There is no obvious trend observed between the mean values of Young's modulus (Y), Bulk modulus (B), and shear modulus (K) for any given *A*_2_AgRhCl_6_ (Table S6). For instance, the arithmetic mean values of these moduli were 40.91 (41.28), 38.39 (40.88), and 15.47 (15.50) GPa for Cs_2_AgRhCl_6_ (Rb_2_AgRhCl_6_), respectively, whereas such a trend is altered for the remaining two systems, with the corresponding values of 41.9 (42.01), 38.6 (29.23), and 14.3 (10.62), respectively. The Young's modulus, which is the ratio of stress to strain, and a measure of stiffness, is found to be largest, 41.28 (40.91) GPa, for Cs_2_AgRhCl_6_ (Rb_2_AgRhCl_6_), showing that they are relatively stiffer than K_2_AgRhCl_6_ and Li_2_AgRhCl_6_. This ultimately suggests that the contribution of covalence in chemical bonds is larger in the former two than in the latter two systems since stiffer solids usually feature significant covalent bond character. The bulk modulus B, which quantifies the resistance to fracture and was calculated using the expression B = (C_11_ +2C_12_)/3, shows a clear increasing trend in the series: Cs_2_AgRhCl_6_ (38.39 GPa) > Rb_2_AgRhCl_6_ (40.88 GPa) > K_2_AgRhCl_6_ (41.92 GPa) > Li_2_AgRhCl_6_ (42.01 GPa). This may lead to the interpretation that Cs_2_AgRhCl_6_ is relatively strengthened compared to other systems across the series and could be more resistant to external forces such as pressure and temperature.

From the values of the elastic properties listed in Table 5, it is quite clear that the anisotropy in Young's modulus, linear compressibility, Bulk modulus and Poisson's ratio is very marginal for Cs_2_AgRhCl_6_. This becomes very marked for Li_2_AgRhCl_6_, and the anisotropy increases in the series from Cs^+^ through Rb^+^ to K^+^ to Li^+^.

**Table 5:** Some selected elastic constants of *A*_2_AgRhCl_6_ (*A* = Cs, Rb, K, Li) ^a,b^.

**System**	**Young's modulus**		**Linear compressibility**		**Shear modulus**		**Poisson's ratio**	
	**Y_**min**_/GPa**	**Y_**max**_/GPa**	**β_min_/TPa^**−1**^**	**β_max_/ TPa^**−1**^**	**K_**min**_/GPa**	**K_**max**_/GPa**	**σ_min_**	**σ_max_**
Cs_2_AgRhCl_6_	40.73	41.17	8.68	8.68	15.39	15.58	0.32	0.33
	1.01		1.00		1.01		1.03	
Rb_2_AgRhCl_6_	34.54	53.37	8.15	8.15	12.71	20.81	0.20	0.49
	1.55		1.00		1.64		2.45	
K_2_AgRhCl_6_	28.40	59.07	7.95	7.95	10.24	23.35	0.15	0.59
	2.08		1.0		2.28		4.05	
Li_2_AgRhCl_6_	15.07	64.73	7.93	7.93	5.23	26.04	0.07	0.78
	4.30		1.0		4.98		11.17	

a *Each second line entry represents the extent of anisotropy*.

b *Subscripts min and max in Y, β, K, and σ represent minimum and maximum values, respectively*.

The empirical measures of brittle/ductile response of mechanical solids are Pugh's criterion (K/B ratio) (Pugh, 1954) and the Cauchy pressure C_P_ (C_P_ = C_12_ – C_44_ for cubic crystals). (Johnson, 1988; Kamran et al., 2009). Materials with K/B > 1.75 are ductile while those with K/B < 1.75 are brittle (Pan and Guan, 2017; Singh et al., 2018). Our calculation gave a K/B value of 2.48 and 2.64 for Rb_2_AgRhCl_6_ and Rb_2_AgRhCl_6_, respectively, and a C_P_ value of 12.6 and 14.3 GPa, respectively. The former property unequivocally suggests the ductile nature of these materials, and the positive nature of C_P_ is expected of any ductile metals, such as Ni or Al, for example (Kamran et al., 2009; Ivanovskii, 2012). This may be consistent with the observation that materials that feature metallic bonding exhibit a positive Cauchy pressure (Thompson and Clegg, 2018). From the mean values of Poisson's ratio σ (values between 0.32 and 0.38 in Table S6), it is obvious that the chemical bonding between metal ions and Cl is increasingly ionic across the series from Cs_2_AgRhCl_6_ to Rb_2_AgRhCl_6_ to K_2_AgRhCl_6_ to Li_2_AgRhCl_6_, given that σ is close to 0.1 – 0.28 for covalent materials (Haines et al., 2001).

## Conclusion

This study used density functional theory to describe the nature of the geometric stability, electronic, transport, optical, and dynamic lattice properties of the series *A*_2_AgRhCl_6_ (*A* = Li, Na, K, Rb, Cs). Although the bandgap of these systems was close to 0.57–0.65 eV with SCAN-*rVV*10, this was, as expected, appreciably underestimated with the traditionally recommended functionals PBE and PBEsol. All these GGA and meta-GGA methods have shown here to be consistently underestimated the bandgaps compared to the quasi-particle GW and hybrid functional HSE06/PBE0 methods, which predict the bandgaps in the visible region, revealing possible application of the studied systems in optoelectronics.

The use of the Global Instability Index suggested that Cs_2_AgRhCl_6_, Rb_2_AgRhCl_6_, and K_2_AgRhCl_6_ might possess perovskite-quality face-centered cubic structures. This was consistent with what emerged from the recommended combination of Goldsmith's tolerance and octahedral factors. However, with the application of the newly-proposed tolerance factor, the first three heavier members of the series were identified as perovskites and the remaining two lighter members as significantly unstable structures. Nevertheless, all showed nearly similar band structure, DOS, and bandgap features. It was shown that the CBM and VBM of *A*_2_AgRhCl_6_ originated mainly from the 4d states of the octahedron Rh^3+^ ion, with a non-negligible contribution from the 3p states of the 6-coordinate Cl^−^ ions.

The reasonably small effective masses of the charge carriers gave evidence of the presence of (quasi) bipolar conductivity and high mobility. These properties, together with the impressive electronic transition features in the dielectric function spectra, high refractive indices, and high absorption coefficients have enabled to us to conclude that the materials studied may be suitable for application in optoelectronics. However, the phonon and elastic properties examined in this study showed that *A*_2_AgRhCl_6_ (A = Li, Na, K, Rb) were dynamically unstable and mechanically stable. This was not the case for Cs_2_AgRhCl_6_, which was predicted to be both dynamically and mechanically stable; this suggests its experimental synthesis and an exploration of its properties appears to be justified.

## Data Availability Statement

The raw data supporting the conclusions of this article will be made available by the authors, without undue reservation.

## Author Contributions

PV: conceptualization, problem design, investigation, literature survey, supervision, and writing—original draft. PV and HM writing—review and editing. All authors contributed to the article and approved the submitted version.

## Conflict of Interest

The authors declare that the research was conducted in the absence of any commercial or financial relationships that could be construed as a potential conflict of interest.
