# Supplementary material for: The Cs2AgRhCl6 Halide Double Perovskite: A Dynamically Stable Lead-Free Transition-Metal Driven Semiconducting Material for Optoelectronics
Source: Front Chem. 2020 Oct 28;8:796. doi: 10.3389/fchem.2020.00796 (PMC7655969; doi:10.3389/fchem.2020.00796)
Supplement: Supplementary file 1 [file Data_Sheet_1.docx]

**The Cs_2_AgRhCl_6_ Double Perovskite: A Dynamically Stable Lead-Free Transition-Metal Driven Semiconducting Material for Optoelectronics**

Pradeep R. Varadwaj,^1,2^* Helder M. Marques^2^

^1^Department of Chemical System Engineering, School of Engineering, The University of Tokyo 7-3-1, Tokyo 113-8656, Japan

^2^Molecular Sciences Institute, School of Chemistry, University of the Witwatersrand, Johannesburg, 2050 South Africa.

Email: [prv.aist@gmail.com](mailto:prv.aist@gmail.com); [pradeep@t.okayama-u.ac.jp](mailto:pradeep@t.okayama-u.ac.jp)

**Supplementary Information**

**Table S1 |** Comparison of selected properties of *A*_2_AgRhCl_6_ (*A* = Li, Na, K, Rb, Cs), evaluated with PBEsol and PBE with SCAN-*rVV*10. Unit of any specific property is same as that given in Table 1. The relaxed conventional cells used.

| System | PBEsol | | | | PBE | | | | SCAN-*rVV*10 | | | |  |
| --- | --- | --- | --- | --- | --- | --- | --- | --- | --- | --- | --- | --- | --- |
|  | *a* | V | GII | E_g_ | *a* | V | GII | E_g_ | *a* | V | GII | E_g_^a^ | Nature of E_g_ |
| Cs_2_AgRhCl_6_ | 10.033 | 1010.0 | 0.123 | 0.42 | 10.316 | 1097.8 | 0.157 | 0.48 | 10.087 | 1026.2 | 0.121 | 1.14 | Direct at *X* |
| Rb_2_AgRhCl_6_ | 9.963 | 988.9 | 0.148 | 0.43 | 10.173 | 1052.8 | 0.139 | 0.50 | 9.961 | 988.2 | 0.149 | 1.17 | Direct at *X* |
| K_2_AgRhCl_6_ | 9.875 | 963.1 | 0.185 | 0.46 | 10.096 | 1029.1 | 0.138 | 0.51 | 9.894 | 968.6 | 0.168 | 1.19 | Direct at *X* |
| Na_2_AgRhCl_6_ | 9.280 | 799.1 | 0.615 | 0.66 | 9.399 | 830.4 | 0.531 | 0.71 | 9.303 | 805.2 | 0.596 | 1.31 | Direct at *X* |
| Li_2_AgRhCl_6_ | 9.759 | 929.4 | 0.431 | 0.49 | 9.952 | 985.7 | 0.460 | 0.55 | 9.805 | 942.5 | 0.422 | 1.22 | Direct at *X* |

^a^ SCF bandgap

**Table S2 |** Comparison of bandgaps (E_g_/eV) predicted using meta-GGA SCAN-*rVV*10 with PBE0, HSE06 and GW methods for *A*_2_AgRhCl_6_ (*A* = Li, K, Rb, Na, Cs).

| System | SCAN-*rVV*10^a^ | SCAN-*rVV*10 ^b^ | PBE0^c^ | HSE06 | G0W0^c^ | GW0^d^ | Nature of *E*_g_ |
| --- | --- | --- | --- | --- | --- | --- | --- |
| Cs_2_AgRhCl_6_ | 1.14 | 0.57 | 3.03 | 2.30 | 2.43 | 2.73 | Direct at *X* |
| Rb_2_AgRhCl_6_ | 1.17 | 0.61 | 3.05 | 2.31 | 2.46 | 2.77 | Direct at *X* |
| K_2_AgRhCl_6_ | 1.19 | 0.63 | 3.05 | 2.32 | 2.49 | 2.79 | Direct at *X* |
| Na_2_AgRhCl_6_ | 1.28 | 0.65 | 3.06 | 2.33 | 2.46 | 2.77 | Direct at *X* |
| Li_2_AgRhCl_6_ | 1.22 | 0.65 | 3.07 | 2.34 | 2.46 | 2.76 | Direct at *X* |

^a^ E_g_ values (SCF) calculated using single-points at the SCAN-*rVV*10 geometries.

^b^ From non-SCF SCAN-*rVV*10 calculation, in which the charge density generated using the SCF calculation on the SCAN-*rVV*10 geometry was used.

^c^ PBE0 functional used for SCF calculation on the SCAN-*rVV*10 geometry, (https://www.vasp.at/wiki/index.php/Specific_hybrid_functionals).

^d^ SCAN-*rVV*10 functional used for SCF calculation and the bandgap calculated using the G_0_W_0_ and GW_0_ methods; the procedure discussed elsewhere followed (https://www.vasp.at/wiki/index.php/Bandgap_of_Si_in_GW).

**Table S3 |** Spin-polarized (SCAN-*rVV*10) effective masses of electrons and holes obtained using the parabolic fitting of the lower conduction band and upper valence band for *A*_2_AgRhCl_6_ (*A* = Cs, Rb, K, Na, Li).^a^

|  | Compound | E_g_/eV | Nature of E_g_ | Carrier type | Direction | | | |
| --- | --- | --- | --- | --- | --- | --- | --- | --- |
|  |  |  |  |  | X→Γ | X→W | Average | ratio (\|*m_e_**/*m_h_*\|*) |
|  | Cs_2_AgRhCl_6_ | 0.57 | Direct at *X* | *m_h_*/m_0_* | -2.44 | -1.51 | -1.98 |  |
|  |  |  |  | *m_e_*/m_0_* | 0.55 | 0.35 | 0.45 | -0.23 |
|  | Rb_2_AgRhCl_6_ | 0.61 | Direct at *X* | *m_h_*/m_0_* | -1.77 | -1.36 | -1.57 |  |
|  |  |  |  | *m_e_*/m_0_* | 0.59 | 0.34 | 0.47 | -0.30 |
|  | K_2_AgRhCl_6_ | 0.63 | Direct at *X* | *m_h_*/m_0_* | -1.56 | -1.34 | -1.45 |  |
|  |  |  |  | *m_e_*/m_0_* | 0.62 | 0.34 | 0.48 | -0.33 |
|  | Na_2_AgRhCl_6_ | 0.65 | Direct at *X* | *m_h_*/m_0_* | -1.36 | -1.30 | -1.33 |  |
|  |  |  |  | *m_e_*/m_0_* | 0.65 | 0.34 | 0.49 | -0.37 |
|  | Li_2_AgRhCl_6_ | 0.65 | Direct at *X* | *m_h_*/m_0_* | -1.32 | -1.30 | -1.31 |  |
|  |  |  |  | *m_e_*/m_0_* | 0.66 | 0.34 | 0.50 | -0.38 |

^a^ *m_0_* is the rest mass of the electron (9.11 x 10^-31^ kg).

**Table S4 |** Comparison of the six eigenvalues of the C_ij_ matrix (values in GPa), obtained with the finite difference method using SCAN-*rVV*10 based relaxed geometries of *A*_2_AgRhCl_6_ (*A* = Cs, Rb, K, Li). The conventional cells used.

| System | λ_1_ | λ_2_ | λ_3_ | λ_4_ | λ_5_ | λ_6_ |
| --- | --- | --- | --- | --- | --- | --- |
| Cs_2_AgRhCl_6_ | 15.39 | 15.39 | 15.39 | 31.16 | 31.16 | 115.16 |
| Rb_2_AgRhCl_6_ | 12.71 | 12.71 | 12.71 | 41.61 | 41.61 | 122.63 |
| K_2_AgRhCl_6_ | 10.24 | 10.24 | 10.24 | 46.69 | 46.69 | 125.76 |
| Li_2_AgRhCl_6_ | 5.23 | 5.23 | 5.23 | 52.07 | 52.07 | 126.04 |

**Table S5 |** Comparison of the three independent elastic constants of the *C_ij_* matrix (values in GPa), obtained with the finite difference method using SCAN-*rVV*10 based relaxed geometries of *A*_2_AgRhCl_6_ (*A* = Cs, Rb, K, Li). The conventional cells used.^a,b^

| System | *C_11_* | *C_12_* | *C_44_* | *C_12_* + 2*C_44_* | [(*C_11_*-( *C_12_* + 2*C_44_*)]≠0 | [*C_44_* – ((*C_11_* – *C_12_*)/2)] |
| --- | --- | --- | --- | --- | --- | --- |
| Cs_2_AgRhCl_6_ | 59.16 | 28.00 | 15.39 | 58.79 | 0.38 | -0.19 |
| Rb_2_AgRhCl_6_ | 68.62 | 27.01 | 12.71 | 52.42 | 9.84 | -8.10 |
| K_2_AgRhCl_6_ | 73.05 | 26.36 | 10.24 | 46.83 | 14.26 | -13.11 |
| Li_2_AgRhCl_6_ | 76.73 | 24.66 | 5.23 | 35.12 | 17.94 | -20.80 |

^a^ *C_11_* = *C_22_* = *C_33_*; *C_12_* = *C_13_* = *C_23_*; *C_44_* = *C_55_* = *C_66_*

^b^ These properties were not evaluated for Na_2_AgRhCl_6_ due to high computational expanse of finite difference calculations.

**Table S6 |** Comparison of elastic properties of *A*_2_AgRhCl_6_ (*A* = Li, K, Rb, Cs), obtained with the finite difference method using SCAN-*rVV*10 based relaxed geometries. The conventional cells used. (These properties were not evaluated for Na_2_AgRhCl_6_ due to high computational expanse of finite difference calculations).

|  |  | **Cs_2_AgRhCl_6_** |  |  |  |
| --- | --- | --- | --- | --- | --- |
| **Property** | **Unit** | **Voigt** | **Reuss** | **Average** | **Nature** |
| Young Modulus (Y) | (GPa) | 40.91 | 40.91 | 40.91 |  |
| Bulk Modulus (B) | (GPa) | 38.39 | 38.39 | 38.39 |  |
| Shear Modulus (K) | (GPa) | 15.47 | 15.47 | 15.47 |  |
| P-wave Modulus | (GPa) | 59.01 | 59.01 | 59.01 |  |
| Poisson ratio (σ) |  | 0.32 | 0.32 | 0.32 |  |
| Bulk/Shear ratio (B/K) |  | 2.48 | 2.48 | 2.48 | (ductile) |
|  |  | **Rb_2_AgRhCl_6_** |  |  |  |
|  |  |  |  |  |  |
| Young Modulus (Y) | (GPa) | 42.34 | 40.22 | 41.28 |  |
| Bulk Modulus (B) | (GPa) | 40.88 | 40.88 | 40.88 |  |
| Shear Modulus (K) | (GPa) | 15.95 | 15.05 | 15.50 |  |
| P-wave Modulus | (GPa) | 62.14 | 60.95 | 61.54 |  |
| Poisson ratio (σ) |  | 0.33 | 0.34 | 0.33 |  |
| Bulk/Shear ratio (B/K) |  | 2.56 | 2.72 | 2.64 | (ductile) |
|  |  | **K_2_AgRhCl_6_** |  |  |  |
|  |  |  |  |  |  |
| Young Modulus (Y) | (GPa) | 41.35 | 35.85 | 38.60 |  |
| Bulk Modulus (B) | (GPa) | 41.92 | 41.92 | 41.92 |  |
| Shear Modulus (K) | (GPa) | 15.48 | 13.20 | 14.34 |  |
| P-wave Modulus | (GPa) | 62.56 | 59.52 | 61.04 |  |
| Poisson ratio (σ) |  | 0.34 | 0.36 | 0.35 |  |
| Bulk/Shear ratio (B/K) |  | 2.71 | 3.18 | 2.92 | (ductile) |
|  |  | **Li_2_AgRhCl_6_** |  |  |  |
|  |  |  |  |  |  |
| Young Modulus (Y) | (GPa) | 36.71 | 21.74 | 29.23 |  |
| Bulk Modulus (B) | (GPa) | 42.01 | 42.01 | 42.01 |  |
| Shear Modulus (K) | (GPa) | 13.55 | 7.69 | 10.62 |  |
| P-wave Modulus | (GPa) | 60.08 | 52.27 | 56.17 |  |
| Poisson ratio (σ) |  | 0.35 | 0.41 | 0.38 |  |
| Bulk/Shear ratio (B/K) |  | 3.10 | 5.46 | 3.96 | (ductile) |

**Table S7 |** Comparison of HSE06, PBE0 and SCAN-*rVV*10 calculated non-spin polarized lattice and bandgap properties of Cs_2_AgRhCl_6_ (space group *Fm*$\bar{3}$*m*) obtained from this work with those of similar halide double perovskites reported experimentally. The lattice properties of the halide double perovskites were relaxed with SCAN-*rVV*10, in conjunction with *k*-mesh 8×8×8. Each second line entry refers to the SCAN-*rVV*10 relaxed properties, whereas the values in parentheses represent non-SCF (fundamental) bandgaps. For HSE06 and PBE0, the *k*-mesh 4×4×4 was used.

| Compound | *a*=*b*=*c*/Å | *V*/Å^3^ | Density(ρ)/gcm^-3^ | E_g_/eV | E_g_/eV(HSE06)^i^ | E_g_/eV(PBE0)^i^ | E_g_/eV(SCAN-*rVV*10)^i^ |
| --- | --- | --- | --- | --- | --- | --- | --- |
| Cs_2_AgInCl_6_^a^ | 10.481  10.378 | 1151.218  1117.66 | 4.05  4.17 | 3.23 | 2.57^l^  2.54 | 2.9-3.3^f^  3.23 | ---  1.34 (0.53) |
| Cs_2_AgBiCl_6_^b^ | 10.777  10.667 | 1251.636  1210.33 | 4.22^p^  4.37 | 2.77^f^ | 2.60–3.15^n^  2.90 | ---  3.57 | ---  1.94 (1.23)^j^ |
| Cs_2_AgSbCl_6_^c^ | 10.701  10.561 | 1225.364^h^  1177.79 | 3.84  3.99 | 2.24 – 2.61^g^ | 2.35^m^  2.28 | ---  2.93 | ---  1.43 (0.88) |
| Cs_2_AgTlCl_6_^d^ | 10.560  10.496 | 1177.420  1156.36 | 4.46  4.54 | 1.96 | 1.09^o^  0.82 | ---  1.50 | ---  ---^k^ |
| Cs_2_AgRhCl_6_^e^ | 10.087 | 1026.200 | 4.46 | --- | 2.30 | 3.02 | 1.14 (0.58) |

^a^ Zhou *et al.*, J. Mater. Chem. A, 2017, 5, 15031-1503

^b^ McClure *et al.*, Chem. Mater. 2016, 28, 5, 1348–1354.

^c^ Zhou *et al.*, J. Mater. Chem. A 2018,6, 2346-2352.

^d^ Slavney *et al*, Angew Chem. Int. Ed. 2018, 57, 12765-12770.

^e^ This work.

^f^ Volonakis *et al* reported E_g_ values between 2.2 and 2.8 eV [*J. Phys. Chem. Lett.* 8, 772-778].

^g^ [Dahl *et al*, Chem. Mater. 2019, 31, 9, 3134–3143] reported bandgaps of 2.823 and 4.15 eV using Tauc plots of dilute nanocrystal solutions for Cs_2_AgSbCl_6_ and of Cs_2_AgInCl_6_, respectively.

^h^ Tran *et al* reported values of 10.664, 1212.7195, 3.878 and 2.54 for *a*, *V*, ρ and E_g_, respectively [Mater. Horiz., 2017,4, 688-693]

^i^ E_g_ values obtained using (SCF) single-points at the SCAN-*rVV*10 geometries (this work), similarly as discussed elsewhere for other systems [Bartel et al, J.Am.Chem.Soc.2020, 142, 5135−5145].

^j^ The PBEsol bandgap was reported to be 1.71 eV [Feng *et al*, J. Phys. Chem. C 2017, 121, 4471−4480]

^k^ SCAN-*rVV*10 yielded a spurious band crossing and the resulting band structure was metallic.

^l^ HSE+SOC [Cia *et al*, Chem. Mater. 2019, 31, 15, 5392–5401].

^n^ HSE06+SOC [Gill *et al*, May 2020, <https://arxiv.org/abs/2006.00183>], in which, the Hartree-Fock exchange fraction and other parameters were adjusted.

^m^ Overall bandgap calculated using direct and indirect bandgaps (Direct bandgap = 3.33 eV; Indirect bandgap = 2.35 eV) [Zhou *et al.*, J. Mater. Chem. A, 2017, 5, 15031-1503].

^o^ HSE06 [Slavney *et al*, Angew Chem. Int. Ed. 2018, 57, 12765-12770].

^p^ We used the atomic information in the crystallographic information file [McClure *et al.*, Chem. Mater. 2016, 28, 5, 1348–1354] reported for Cs_2_AgBiCl_6_ to calculate the density.


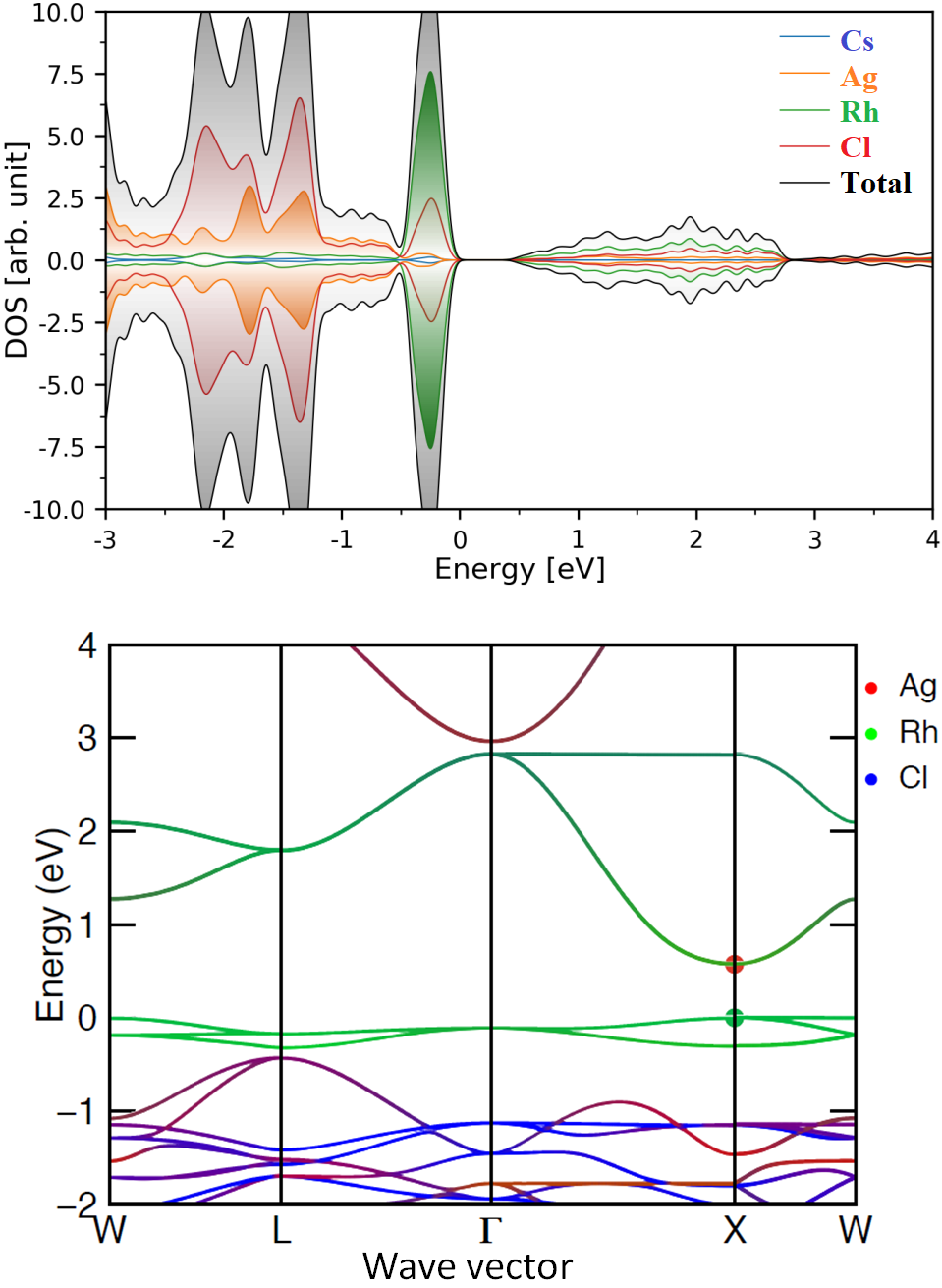


**FIGURE S1 |**  Calculated spin-polarized electronic density of states (Top) and band structure (bottom) for Cs_2_AgRhCl_6_ double perovskite. The primitive cell used.


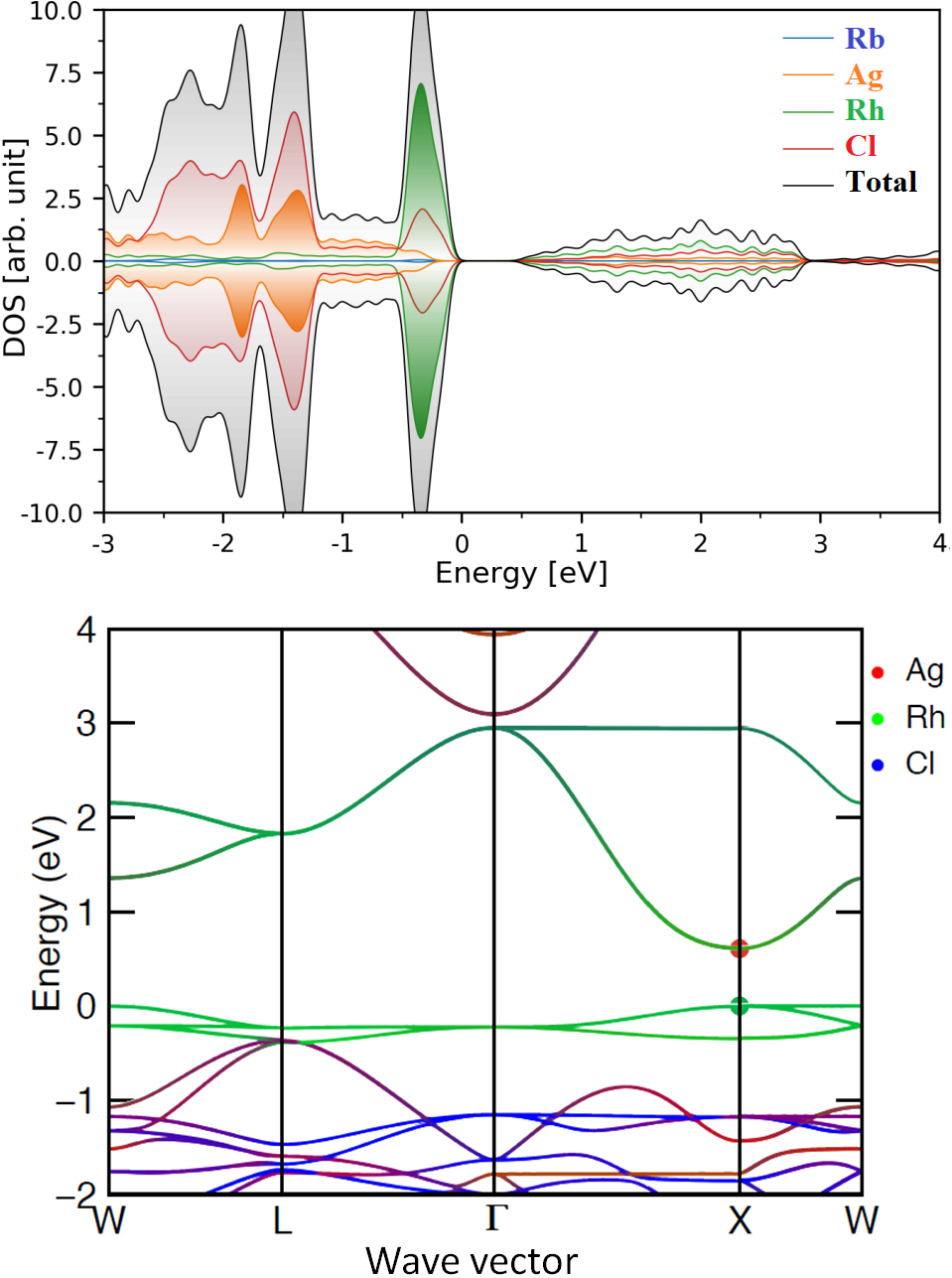


**FIGURE S2 |** Calculated spin-polarized electronic density of states (Top) and band structure (bottom) for Rb_2_AgRhCl_6_ double perovskite. The primitive cell used.


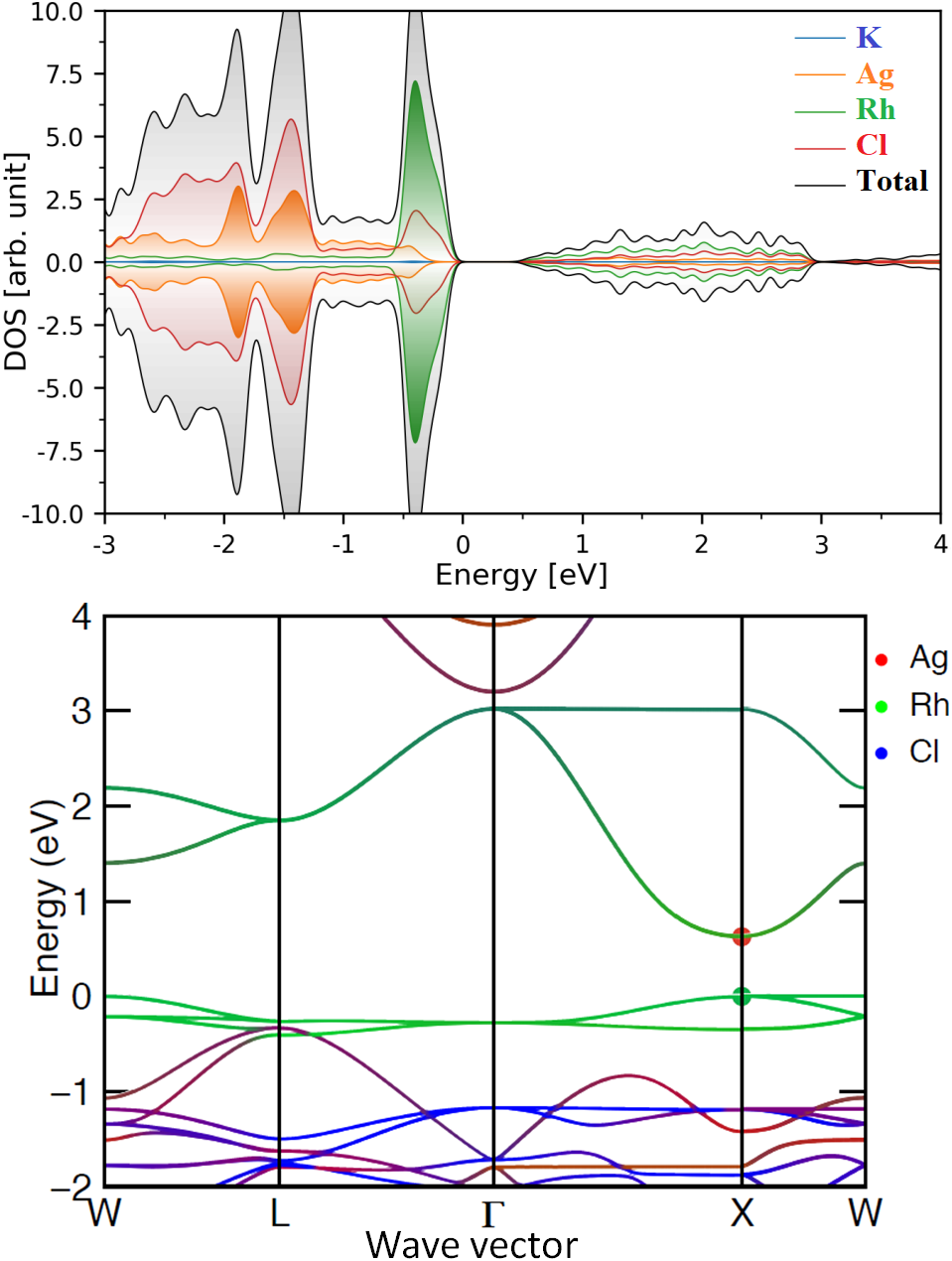


**FIGURE S3 |** Calculated spin-polarized electronic density of states (Top) and band structure (bottom) for K_2_AgRhCl_6_ double perovskite. The primitive cell used.


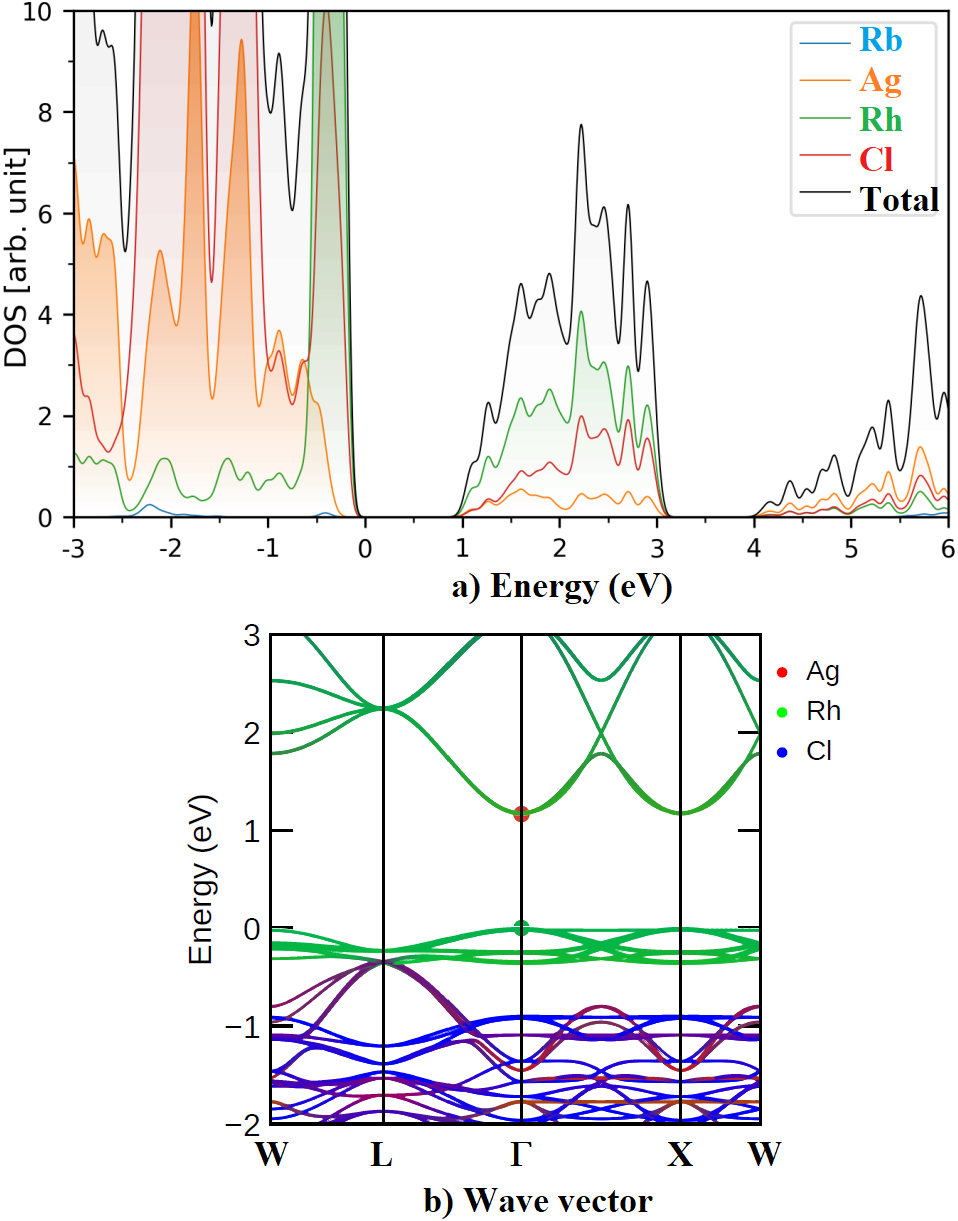


**FIGURE S4 |** a) Calculated atom-projected density of states and b) electronic band dispersion for Rb_2_AgRhCl_6_ double perovskite. The conventional cell used.


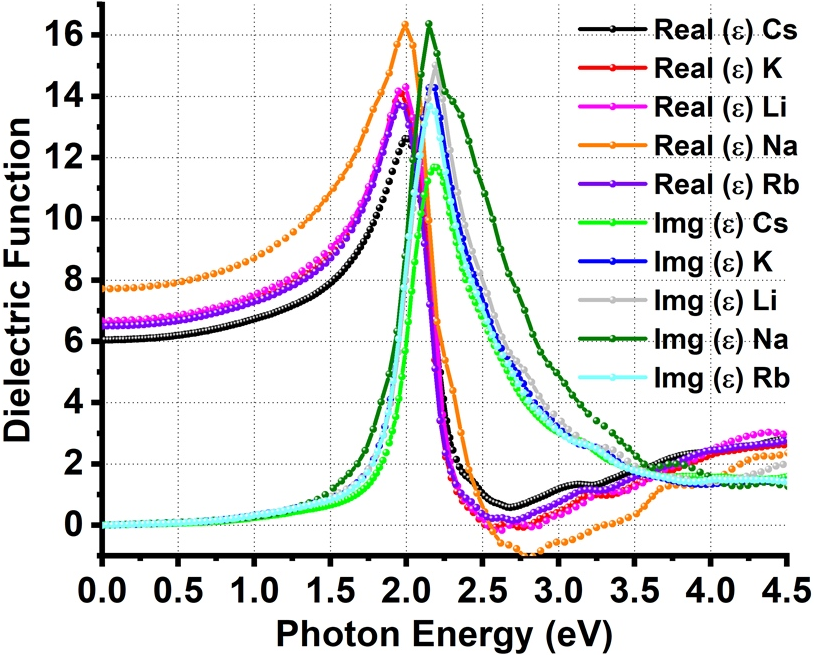

**FIGURE S5 |** (Top) Dependence of the real and imaginary parts of dielectric function *ε*(*ω*) on the photon energy for *A*_2_AgRhCl_6_ (A = Li, K, Na, Rb, Cs). The conventional unit-cells, together with the k-mesh (10×10×10), used. (Bottom) Dependence of the a) absorption coefficient, b) reflectivity, d) energy-loss function on the photon energy for the corresponding systems. Displayed in c) is the Tauc plot for the same systems. Spin-polarized calculations adopted.


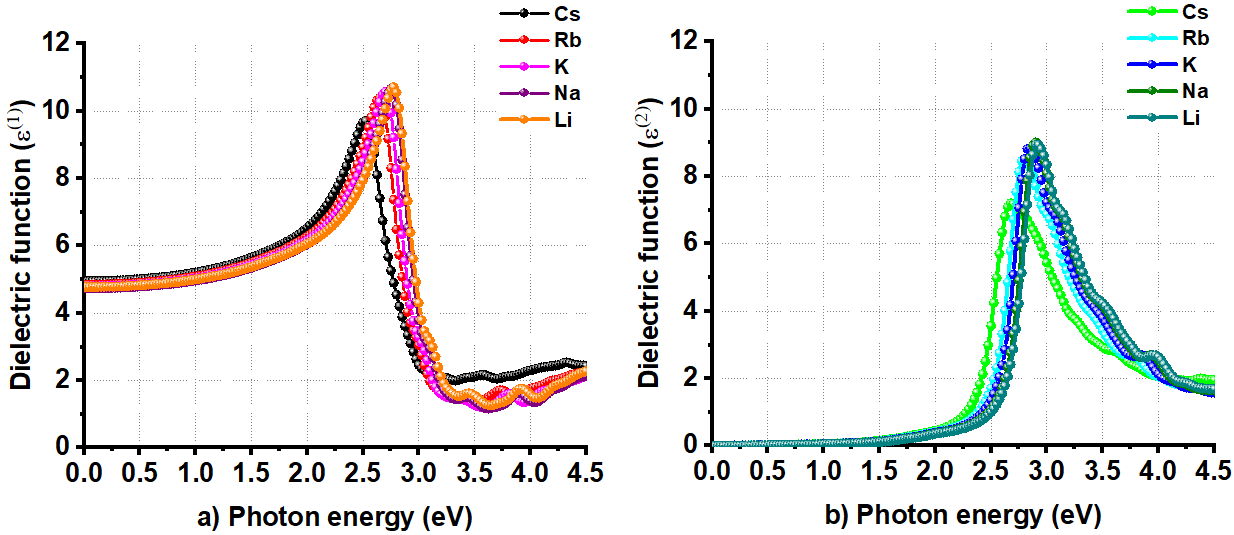


**
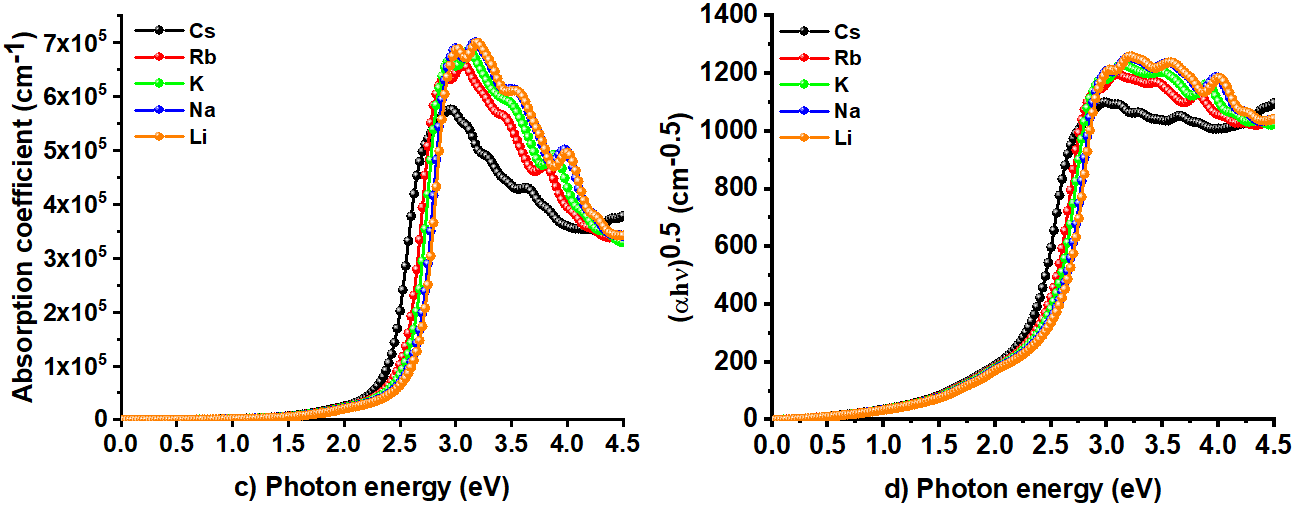
**

**FIGURE S6 |** Dependence of the a) real and b) imaginary parts of dielectric function *ε*(*ω*) on the photon energy for *A*_2_AgRhCl_6_ (A = Li, K, Na, Rb, Cs), obtained using SCAN-*rVV*10 (k-mesh 12×12×12). Included in c) is the dependence of absorption coefficient on the photon energy, and in d) the Tauc plot. The SCAN-*rVV*10 relaxed geometries of primitive unit-cells were used. Non-spin polarized calculations were adopted.


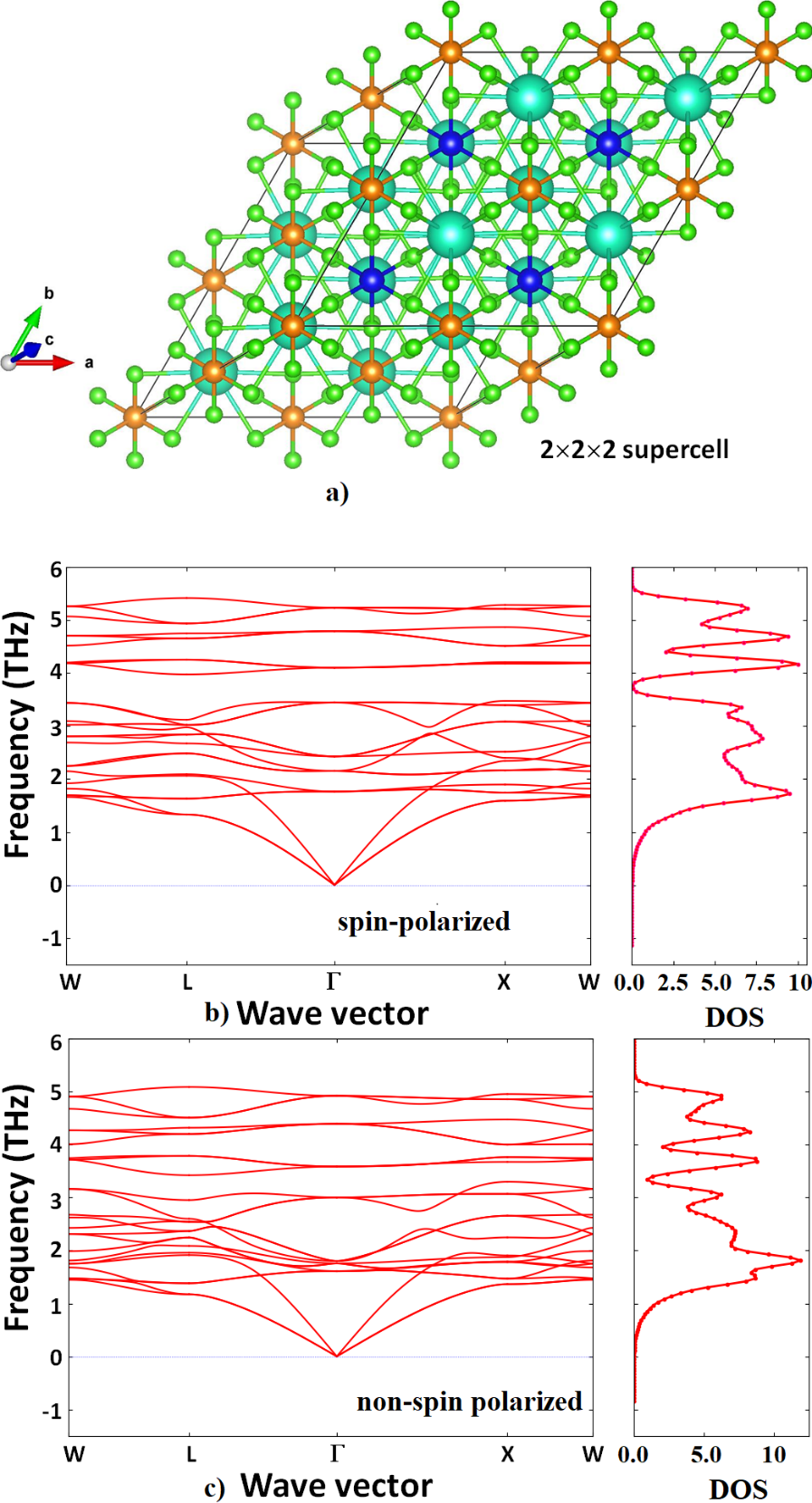


**FIGURE S7 |** Phonon dispersion and total density of states for Cs_2_AgRhCl_6_ (bottom), calculated with b) the finite-difference and c) DFPT methods using the (2×2×2) supercell shown in a). (see Fig. 1 illustrates for atom types). The supercell was built using the primitive unit-cell of the system.

**FIGURE S7 |** Calculated phonon dispersion of *A*_2_AgRhCl_6_ (*A* = Cs, Rb) double perovskites, showing prominent phonon frequency modes below 10 THz (within the harmonic approximation).

~~
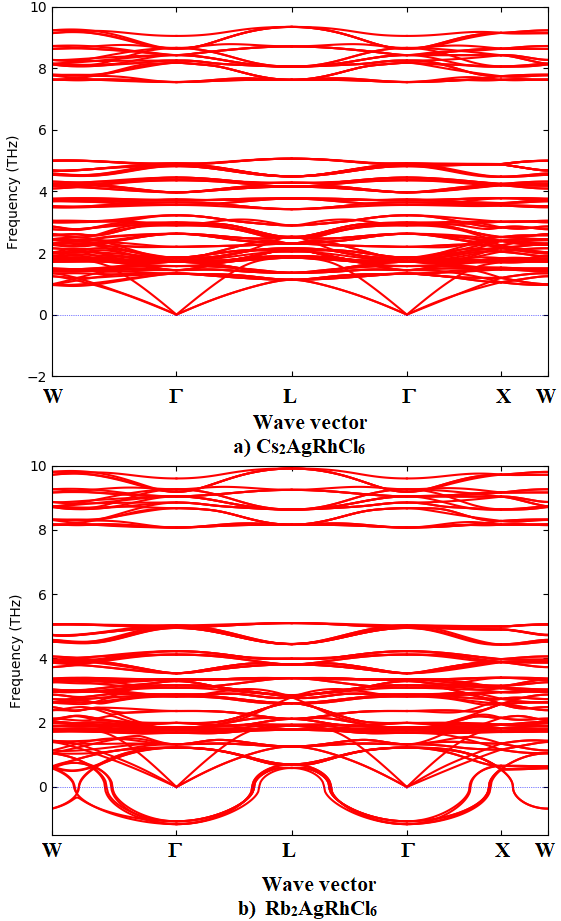
~~

**FIGURE S8 |** Calculated phonon dispersion of *A*_2_AgRhCl_6_ (*A* = Cs, Rb) double perovskites, showing prominent phonon frequency modes below 10 THz (within the harmonic approximation). The conventional unit-cells used.


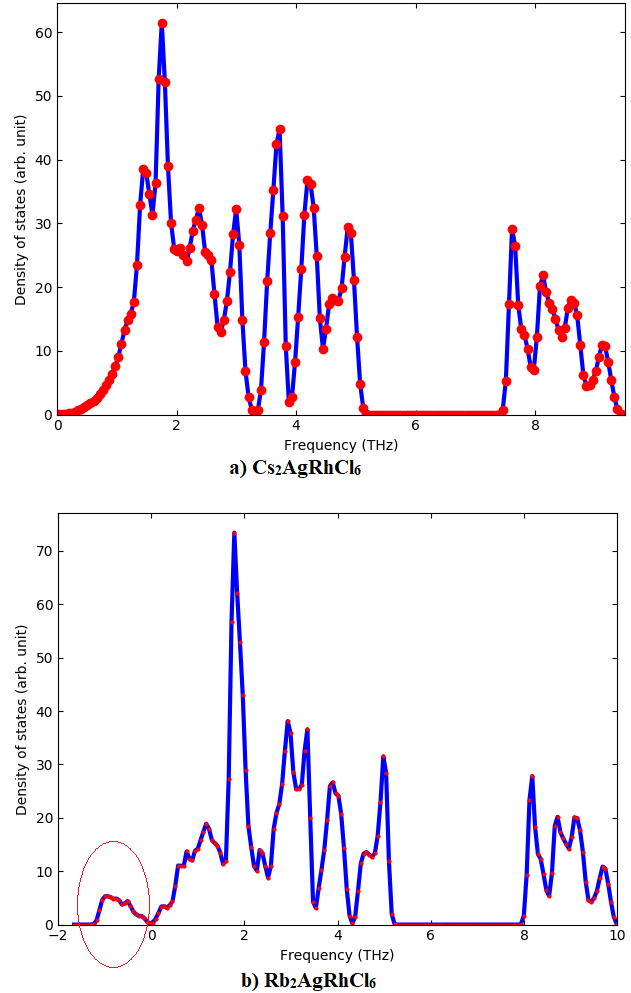


**FIGURE S9 |** Phonon density of states (Total) for *A*_2_AgRhCl_6_ (*A* = Cs, Rb) double perovskite, showing phonon frequencies below ≤ 10 THz. The part of the spectrum encircled by an ellipse in the low frequency region in b) refers to imaginary frequency. The conventional unit-cells used.
